# Postcranial anatomy of the Late Miocene Eurasian hornless rhinocerotid *Chilotherium*

**DOI:** 10.1371/journal.pone.0336590

**Published:** 2025-12-05

**Authors:** Panagiotis Kampouridis, Georgia Svorligkou, Nikolai Spassov, Madelaine Böhme

**Affiliations:** 1 Terrestrial Palaeoclimatology section, Department of Geosciences, Eberhard Karls University of Tübingen, Tübingen, Germany; 2 Palaeontology Section, Senckenberg Centre for Human Evolution and Palaeoenvironment, Tübingen, Germany; 3 Faculty of Geology and Geoenvironment, Department of Historical Geology and Palaeontology, National and Kapodistrian University of Athens, Athens, Greece; 4 National Museum of Natural History at the Bulgarian Academy of Sciences, Sofia, Bulgaria; Universita degli Studi della Basilicata, ITALY

## Abstract

The hornless rhinocerotid genus *Chilotherium* is one of the most common rhinoceroses found in Eurasia during the Late Miocene, even being the most abundant large mammal in some localities. Despite its high frequency, much is still unknown about the anatomy and taxonomy of some representatives of the group, because most studies focus solely on cranial and dental remains. For this reason, we describe the postcranial material of the three well-known chilothere species *Chilotherium persiae* from the Upper Miocene of Maragheh (Iran), *Chilotherium habereri* from the Upper Miocene of Kutschwan (China), and *Chilotherium schlosseri* from the Upper Miocene of Samos (Greece). A comparison to postcranial material of other chilotheres from the literature reveals some characters that can assist the identification of some species, such as the morphology and dimensions of the patella and the metapodials, the morphology of the proximal articulation of tibia, and the arrangement of the articular facets for the calcaneum on the astragalus. The comparison supports previous hypotheses about the relationships of some chilotheres that were only based on cranial features, including the plesiomorphic nature of ‘*Chilotherium*’ *wimani* from the Upper Miocene of China and the close relationship of *C. persiae* and *C. habereri*.

## Introduction

Rhinoceroses are amongst the most iconic large mammals to roam the earth today. Even though only five extant species exist, in the past this group was much more diverse [1–6]. During the Miocene a plethora of rhinoceros species existed and, although the extant representatives are characterised by the presence of horns on their heads, a highly diverse rhinocerotid group were the aceratheriines, also known as the hornless rhinos. One of the most characteristic and species-rich genera of this group was *Chilotherium* Ringström, 1924 [7] that lived in Asia and Eastern Europe during the Late Miocene [1,8,9]. This genus is characterised by a very wide mandibular symphysis with two large, tusk-like incisors, flat to concave frontal bones, very complex enamel folds in the upper teeth, and the appendicular skeleton is notably shortened and relatively robust, especially the metapodials [7,10].

Ringström (1924) [7] initially attributed over ten species to this newly erected genus, while also erecting several new species, based on material from the Upper Miocene of China. He discussed that the representatives of *Chilotherium* were extremely similar and that they could only be separated based on the morphology of the upper teeth. However, it has been shown that the morphology of the skull over all is more informative for the taxonomic attribution than the tooth morphology [e.g., 10,11]. Some studies actually suggested that also the postcranial anatomy of some species can be used for their distinction [11,12]. However, only few studies have reported postcranial material for members of this group [e.g., 7,13–15] and even less studies described and analysed this material in detail [11,16]. This creates a large gap in our understanding of the body plan of this rhino group and the potential interspecific variability. However, it was suggested early on that the appendicular skeleton of the genus *Chilotherium* is very derived [7], which was also confirmed by later studies, suggesting that the limb bones of chilotheres differ from those of other aceratheriines [3,16]. Korotkevitch [11], Krokos [17], and Deng [16] studied large samples of some chilothere species, suggesting that some elements, such as the metapodials, are also important for taxonomic purposes within the group. These findings have not been verified or re-evaluated by any later study, making some assumptions somewhat ambiguous.

**Fig 1:**
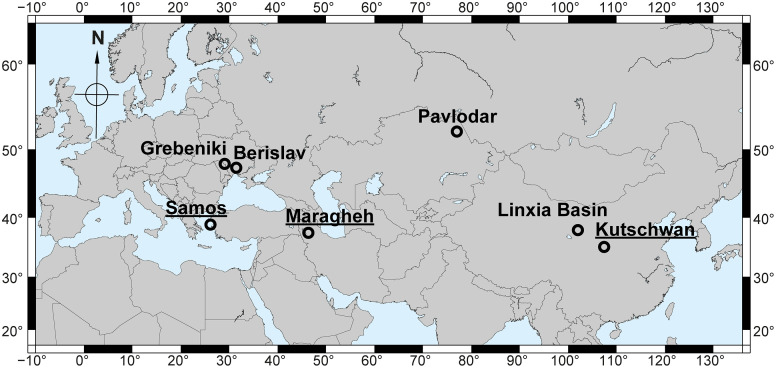
Geographical map with the fossil localities of the compared chilotheres. The localities where the herein studied material comes from (Samos in Greece, Maragheh in Iran, and Kutschwan in China) are underlined. The map was generated using Generic Mapping Tools 6 (GMT6) [18].

The aim of this study is to evaluate the postcranial material of *Chilotherium* spp. available in different institutions to provide some insight into the taxonomy and interrelationships of this group. For this purpose, we studied collections of three historical fossil localities, Maragheh (Iran), Kutschwan (China), and Samos (Greece), that are known to include *Chilotherium* in their fauna (Fig 1). The studied specimens were compared with available material in the literature from Europe and Asia (Fig 1), thereby covering almost the complete geographical distribution of this taxon and offering new insight into the anatomy and taxonomy of these animals.

## Abbreviations

### Anatomical abbreviations

APD, anteroposterior diameter; APDbec, anteroposterior diameter measured at the level of the posterior astragalar facet; APDcaput, anteroposterior diameter of the humerus head; APDdia, anteroposterior diameter of the diaphysis; APDdist, distal anteroposterior diameter; APDdist art, anteroposterior diameter of the distal articular surface; APDinf, medial anteroposterior diameter; APDolecr, anteroposterior diameter of the olecranon; APDprox, proximal anteroposterior diameter; APDprox art, anteroposterior diameter of the proximal articular surface; APDsommet, anteroposterior diameter of the tuber calcis; DL, distance between the lips of the trochlea; TD, transversal diameter; TDcaput, transversal diameter of the humerus head; TDdia, transversal diameter of the diaphysis; TDdist artic, transversal diameter of the distal articular surface; TDdist max, maximal distal transversal diameter; TD maxi dist, maximal distal transversal diameter; TDmini post, minimal transversall diameter; TDolecranon, transversal diameter of the olecranon; TDprox art, transversal diameter of the proximal articular surface; TDprox max, maximal proximal transversal diameter; TDsommet, transversal diameter of the tuber calcis; TDsust, maximal transversal diameter at the sustentacular tali; H, height; Hant, anterior height; Hface ant, height of anterior facet; H lateral condyle, height of the lateral distal condyle; H medial condyle, height of the medial distal condyle; Hpost, posterior height measured between the tubers calcis and the posterior astragalar facet; Htuberosity, height of the lateral tuberosity; L, length; Lmax, maximal length; L absolute, maximal length; L anatom, length in anatomical position; Lart sup, length of the anterior articular surface; Lart inf, length of the central articular surface; Lcaput, length measured from the humerus head; l, width; lart, width of articular surface; and lart sup, width of anterior articular surface.

### Institutional abbreviations

AMNH, American Museum of Natural History, New York (USA); AMPG, Athens Museum of Palaeontology and Geology of the National and Kapodistrian University of Athens (Greece); BSPG, Bayerische Staatssammlung für Paläontologie und Geologie, Munich (Germany); GMM, Geomuseum of the University of Münster (Germany); GPIT, Geologisch-Paläontologisches Institut der Universität Tübingen (Germany); GPIH, Geologisch-Paläontologisches Institut der Universität Hamburg (Germany); HLMD, Hessisches Landesmuseum Darmstadt (Germany); IPUW, Institut für Paläontologie, Universität Wien (Austria); MLU, Martin-Luther Universität Halle-Wittenberg (Germany); MNHN, Muséum national d’Histoire naturelle, Paris (France); NHMW, Naturhistorisches Museum in Wien (Austria); NMB, Naturhistorisches Museum Basel (Switzerland); and SMNS, Staatliches Museum für Naturkunde Stuttgart (Germany); and SNSB, Staatliche Naturwissenschaftliche Sammlungen Bayerns (Germany).

## Materials and methods

For the purpose of this study, material of three distinct chilotheres was studied: *Chilotherium persiae* (Pohlig, 1885) [19] from the Upper Miocene deposits of Maragheh in Iran [13,20–23], *Chilotherium habereri* (Schlosser, 1903) [24] from the Upper Miocene deposits of Kutschwan in China [25,26], and *Chilotherium schlosseri* (Weber, 1905) [14] from the Upper Miocene deposits of Samos in Greece [8,27–29] (Fig 1). The material studied herein is housed in different palaeontological collections (GMM, GPIT, IPUW, MNHN, NHMW, and MLU).

Additionally, the studied material is compared to published data from the species ‘*Chilotherium*’ *wimani* Ringström, 1924 [7] from the Upper Miocene of China [16], *Chilotherium kowalevskii* (Pavlow, 1913) [15], *Chilotherium sarmaticum* Korotkevitch, 1958, both from the Upper Miocene of Ukraine [11,15,17], and *Chilotherium orlovi* Bayshashov, 1982 [30] from the Upper Miocene of Pavlodar in Kazakhstan [12,30].

Measurements were taken according to Guérin (1980) [31] with some additions. Detailed measurements of all bones from the literature and those measured herein are provided in S1 Tables 1–23 and throughout the main text in the Tables. The anatomical terms used in this study are based on the literature [31–35]. It has to be noted that in the older literature [7,11,12,14,17] no standardised measuring protocol like that of Guérin (1980) [31] has been used. Therefore, it is difficult to make certain and detailed comparisons with some of the species. This is especially true for complex bones like carpals and tarsals, where it is even more difficult to compare measurements if they did not follow the exact same method of measuring.

### Taxonomic notes

The taxonomy of chilotheres has been a rather complicated matter for a long time [8,10,17,36–38]. The genus was erected over 100 years ago [7] and over a dozen species have been assigned to it over the years, with many attributions not being valid anymore (see [7,37–39]). Especially the European representatives have been the centre of great confusion, with the erection of overall 10 species, two of which come from Portugal and in fact belong to the elasmotheriine rhinoceros *Hispanotherium* [37,38]. All other European chilotheres come from Eastern and Southeastern Europe and are validly assigned to the chilotheres. The taxonomy of these other eight species, while representing chilotheres, has been a complicated matter to this day. From the Upper Miocene of Samos alone, four species have been erected, two of which, *Chilotherium wegneri* (Andree, 1921) [40] and *Chilotherium angustifrons* (Andree, 1921) [40], have subsequently been synonymised to *C. schlosseri* [8]. *Chilotherium schlosseri* and the fourth species, *Eochilotherium samium* (Weber, 1905), were recently revised and separated on a generic level, with both species belonging to the subtribe Chilotheriina (sensu [10]). The other chilothere species from Greece is *Aceratherium kiliasi* Geraads and Koufos, 1990 [41], which was erected based on craniomandibular material from the Upper Miocene of Pentalophos in northern Greece. The material from Pentalophos belongs in part to the non-chilothere aceratheriine *Acerorhinus neleus* Athanassiou et al., 2014 [42]. The holotype and some other specimens, do belong to a chilothere, but represent the species *E. samium*, making *Aceratherium kiliasi* a junior synonym of this species [10,42]. The other European chilotheres were all erected based on material from Ukraine. A partial skull from Upper Miocene deposits near Odessa represents the holotype of *Teleoceras ponticus* Lubicz-Niezabitowski, 1912 [43,44]. Shortly after its’ erection it was suggested to represent a junior synonym of *C. schlosseri* [36] and is still considered as such [8]. The other two species are *Chilotherium kowalevskii* (Pavlow, 1913) [15] from the Upper Miocene of Grebeniki and *Chilotherium sarmaticum* Korotkevitch, 1958 [45] from the Upper Miocene of Berislav (Ukraine). The first of these two has repeatedly been suggested to be a junior synonym of *C. schlosseri* [11,17,46,47]; however, recent re-evaluations of the Samian chilotheres showed that the two species differ both in their cranial and dental morphology [8,10]. The species *C. schlosseri* features widely separated parietal crests, with a minimal distance of at least 70 mm (n = 7), whereas the material of *C. kowalevskii* from Grebeniki exhibits a value range of 40–66 mm (n = 10) for this measurement, thus clearly differing from *C. schlosseri*. *Chilotherium sarmaticum*, on the other hand, has received little attention after its initial description and was usually neglected in most comparative studies [8,48]. However, it was shown that *C. sarmaticum* differs from both *C. kowalevskii* from Grebeniki and *C. schlosseri* from Samos [10,11]. Therefore, both *C. kowalevskii* and *C. sarmaticum* are herein considered valid species. Regarding the *Chilotherium* species from China, there are several species that were attributed to the genus by Ringström (1924) [7], including some newly erected species and subspecies. Some of these cannot be considered as valid species, while other do not belong to the genus *Chilotherium*. Several studies have tried to bring order into this matter [e.g., 37,38]. Heissig (1975) [38] attempted to distinguish different lineages within the genus *Chilotherium* by proposing subgenera. Only few researchers adopted this scheme [e.g., 48] and it is not used anymore. More recent work on the rhinocerotids from China has shed new light on the issue, separating distinct genera, and for instance erecting new chilothere species, like ‘*Chilotherium*’ *primigenium* Deng, 2006 [37] and *Chilotherium licenti* Sun et al., 2018 [49]. Additionally, it was recently discussed that the two more ‘primitive’ species ‘*C.*’ *primigenium* [37] and ‘*C.*’ *wimani* [7] may in fact not belong to the genus *Chilotherium*. It was discussed that they lack the characteristic depression of the frontal region and exhibit some other more plesiomorphic characters that may associate these two species closer with *E. samium* instead [10]. A detailed re-evaluation of the type material is needed to clarify the generic status of these species. However, for the time being both species will be referred to as ‘*C.’ primigenium* and ‘*C.’ wimani*.

### Fossil sites

#### Maragheh.

The locality of Maragheh is situated in northwestern Iran (Fig 1). The Late Miocene fauna is well known since the late 19^th^ century [19,50,51]. Several fossil sites in the area have been excavated over the last century [13,23,50,52,53], recent studies suggested the existence of three distinct fossiliferous horizons and were able to correlate the old fossil sites with these three horizons [21,22]. Material from Maragheh is scattered throughout many collections, such as the MLU, MNHN, and NHMW. The rhino assemblage in Maragheh is quite rich and includes the huge elasmotheriine *Iranotherium morgani* (Mecquenem, 1908) [50] the large tandem-horned ‘*Ceratotherium*’ *neumayri* (Osborn, 1900) [54], and two smaller aceratheriines, *Chilotherium persiae* and *Persiatherium rodleri* Pandolfi, 2016 [20]. The last species, was only recently described and is mainly known from its holotype – a subadult skull [20]. Maragheh is the type locality of all four of these rhinocerotid species and the first two are also well known from other localities in Eurasia, showing the importance of the locality for rhinocerotid systematics. *Chilotherium persiae* is by far the most common representative of the family in the Maragheh fauna and a rich collection of postcranial material is available for this species. Mecquenem (1924) [13] was the first to provide a more detailed description of *C. persiae* and while shortly describing also some postcranial elements he mainly focussed on the cranial and dental material. The studied material comes from different fossil sites situated in the area north of Maragheh, including ‘Ketschawa’ or Karajabad, Kara Kend, and Kopran, which represent the middle and lower fossiliferous beds of Maragheh [55,56].

As mentioned above, four rhinocerotid species existed in Maragheh and during the course of this project postcranial elements not only of *C. persiae* but also of the other species were found. The horned rhinoceroses ‘*Ceratotherium’ neumayri* and *Iranotherium morgani* are much larger and their postcranial bones are easily distinguishable from *Chilotherium*. The other hornless species, *Persiatherium rodleri*, is basically only known from a single skull, apart from it only some isolated dental and fragmentary postcranial elements from the Upper Miocene of Küçükçekmece (Turkey) have been attributed to *Persiatherium* sp. [46]. However, they are not particularly helpful in distinguishing the species. Based on the phylogenetic analysis of Lu et al. (2023) [57], *Persiatherium rodleri* is placed in Aceratheriinae, but distinct from Chilotheriina, which is the clade made up by *Chilotherium* and *Shansirhinus* in Lu et al. (2023) [57], although not referred to as such by the authors. This further supports the notion that *Persiatherium rodleri* should not exhibit the shortened limbs that are known in *Chilotherium*. All herein reported specimens, fit both metrically and morphological the known postcranial elements of *Chilotherium*.

#### Kutschwan.

The locality of Kutschwan in China is of Late Miocene age and was discovered by the German geographer and physician Albert Tafel in Shanxi during his trip to China in 1905 [25]. No information about the exact location of the fossil site is known, but the material was collected from horizontal red clay deposits close to the Yellow River (Huang He) in Shanxi (China, Fig 1) [25]. The material was excavated by A. Tafel, comprising of many cranial and postcranial elements. The whole collection was initially prepared at the SMNS (Germany) before being deposited in the collections of the GPIT (Germany) and is still housed there today. Hugo Killgus studied the whole collection for his Ph.D. Dissertation [26] and recognised a quite rich mammalian fauna, based on this limited material, including an ictithere hyaena [58], two rhinocerotids, *Chilotherium habereri*, *Parelasmotherium schansiense* Killgus, 1923 [25], a hipparionine horse, the giraffe *Schansitherium tafeli* Killgus, 1923 [25], one large and up to three small bovids [25]. More recently, Kampouridis et al. (2022) [59] reassessed the taxonomy and phylogeny of *Parelasmotherium schansiense* by studying its holotype.

The chilothere *C. habereri* is the most abundant taxon in this collection, including several skulls and a small sample of postcranial material, which cannot be associated with the huge elasmotheriine. The cranial and dental morphology of the species were revised by Ringström (1924) [7] after the original description [24], but not much is known about the postcranial anatomy of the species. The herein reported postcranial elements can only be assigned to the single aceratheriine present in Kutschwan, *C. habereri*.

#### Samos.

The island of Samos (Fig 1) has been known to yield Late Miocene vertebrate fossils since the 19^th^ century [60]. Since then, numerous palaeontologists and fossil hunters have travelled to the island to collect fossils [60]. This led to several impressive collections of Samos material in famous natural history museums throughout the world, including among others the AMNH, NHMW, and SMNS. Most recently, the Aristotle University of Thessaloniki, led by Prof. George Koufos, excavated on Samos Island, bringing to light a rich collection of mammalian remains [60,61]. The material was studied in detail, providing crucial information about the (bio-)stratigraphical context and the fauna itself [e.g., 28,29,61,62]. The rhinocerotid material from Samos has been assigned to several different taxa over the years [8,10,27]. Today, it is generally accepted that two large tandem-horned rhinocerotines are present, ‘*Ceratotherium*’ *neumayri* and *Dihoplus pikermiensis* (Toula, 1906) [63], along with two small aceratheriines, *Chilotherium schlosseri* and *Eochilotherium samium* [10,14,27,28,64]. The taxonomy of the Samian chilotheres has experienced many difficulties in the past, with the suggested presence of five different species depending on the authors – *C. schlosseri*, *E. samium*, *C. wegneri*, *C. angustifrons*, and *C. kowalevskii* [e.g., 8,14,17,27,40,42]. Most recently, Kampouridis et al. (2023) [8,10] suggested that the only valid chilothere species in Samos are *C. schlosseri* and *E. samium*, with *C. wegneri* and *C. angustifrons* representing junior synonyms of *C. schlosseri*. The species *E. samium* is much rarer than *C. schlosseri* and is currently only known from a single skull (SMF M 3601) with its associated mandible from the locality of Samos.

No postcranial material is assignable to *E. samium*, whereas *C. schlosseri* is represented by a large amount of material including, an impressive number of skulls [14,40,65–67] but also postcranial elements, which differ significantly from the much larger ‘*Ceratotherium*’ *neumayri* and *Dihoplus pikermiensis*. This postcranial material is morphologically and metrically much closer to that of typical chilotheres, such as *C. kowalevskii*, *C. anderssoni* Ringström, 1924 [7], and *C. orlovi*, than to the more plesiomorphic ‘*C.*’ *wimani* (see also below); therefore, an assignment to *E. samium* is excluded.

### Systematic Palaeontology

Class Mammalia Linnaeus, 1758 [68]

Order Perissodactyla Owen, 1848 [69]

Family Rhinocerotidae Gray, 1821 [70]

Subfamily Aceratheriinae Dollo, 1885 [71] (sensu Lu et al. (2023) [57])

Tribe Aceratheriini Dollo, 1885 [71] (sensu Lu et al. (2023) [57])

Subtribe Chilotheriina Qiu et al., 1987 [72] (sensu Kampouridis et al. (2023) [10])

### Included genera

*Chilotherium* Ringström, 1924 [7], *Shansirhinus* Kretzoi, 1942 [73], and *Eochilotherium* Geraads and Spassov, 2009 [48].

### Diagnosis

Aceratheriine rhinocerotids that feature the following autapomorphic traits: separated parietal crests; upper molars featuring a marked protocone constriction and a moderate to strong antecrochet that tends to project lingually; a mandible that is characterised by a very wide mandibular symphysis with a flat to concave ventral surface; large, sexually dimorphic i2s that exhibit a trend to become more flattened and tusk-like, especially in males, and are separated from each other by a wide diastema and from the p2 by a long diastema with a marked crest. The group also exhibits an upper ﬁrst deciduous premolar retained into adulthood, whereas the ﬁrst lower deciduous premolar, when present, is shed and not replaced by a permanent one. Additionally, the group lacks any upper incisor and the upper cheek teeth have generally pronounced secondary enamel folds (i.e., crista, crochet, and antecrochet), and the appendicular skeleton is notably shortened and relatively robust, especially the autopodium (modiﬁed after [10]).

### Remarks

The clade Chilotheriini was originally established by Qiu et al. (1987) [72] as a tribe, to encompass the genera *Acerorhinus* and *Chilotherium*, specifically excluding the genus *Aceratherium*. However, the relationship between these two genera is not clear, as shown in the contradicting results of some recent phylogenetic analyses [20,57]. Therefore, it has not been possible to prove that these two genera form a monophyletic clade. In fact, *Acerorhinus* shows many plesiomorphic characters and none of the apomorphic features characterising representatives of *Chilotherium*, therefore a close relationship cannot be supported. *Aceratherium* shows characters that are more similar to *Chilotherium*, like the somewhat shortened limb bones [74], whereas in *Acerorhinus zernowi* the plesiomorphic state of more elongated limb bone is seen [75]. Another more enigmatic hornless rhinocerotid, *Shansirhinus*, is much more similar to *Chilotherium* in many regards, such as the wide mandibular symphysis, the more complicated enamel folds, and the higher tooth crowns [76]. Their probably closer relationship has also been suggested in recent phylogenetic analyses [20,57]. It would therefore be best to remove *Acerorhinus* from Chilotheriina and to restrict this group to the genera *Chilotherium*, *Shansirhinus*, and *Eochilotherium*, with the latter one having been regarded as belonging to *Chilotherium* sensu stricto up until recently [8,10,27].

The type genus of Chilotheriini was not designated as such by Qiu et al. (1987) [72]; however, based on ICZN Arts. 29.1 and 64, a suprageneric name must derive from its type genus, which would naturally make *Chilotherium* the type genus. Following, the recommendation of Kampouridis et al. (2023) [10], we herein use the clade at a subtribe rank (as Chilotheriina) with *Chilotherium* as the type genus and also including the genera *Shansirhinus* and *Eochilotherium*.

Genus *Chilotherium* Ringström, 1924 [7]

### Type species

*Chilotherium anderssoni* Ringström, 1924 [7].

### Included species

*Chilotherium persiae* (Pohlig, 1885) [19], *Chilotherium habereri* (Schlosser, 1903) [24], *Chilotherium schlosseri* (Weber, 1905) [14], *Chilotherium kowalevskii* (Pavlow, 1913) [15], ‘*Chilotherium*’ *wimani* Ringström, 1924 [7], *Chilotherium sarmaticum* Korotkevich, 1958 [45], *Chilotherium orlovi* Bayshashov, 1982 [30], ‘*Chilotherium*’ *primigenium* Deng, 2006 [37], and *Chilotherium licenti* Sun et al., 2018 [49].

### Diagnosis

Aceratheriine rhinocerotids that feature the following autapomorphic characters: flat and wide skull, ﬂattened and depressed frontal region; well-developed postorbital processes; moderately to widely separated parietal crests; highly placed orbits; very wide mandibular symphysis that features a concave ventral side; very large, ﬂattened, tusk-like second lower incisors, with a scalene triangle cross section and upturned, dorsomedially oriented wear facets; reduced premaxillary bones that lack upper incisors; and very strong secondary enamel folds, including a lingually ﬂattened and strongly constricted protocone in the molars. It is also characterised by a relatively short length of the premolars compared with the molars, mainly due to the reduced size of the P2 and p2; and notably shortened metapodials and relative robust appendicular skeleton (modiﬁed after [7,10,27,39,48]).

### Remarks

Recently, it was shown that ‘*C.*’ *wimani* and ‘*C.*’ *primigenium* exhibit more plesiomorphic features than the other *Chilotherium* species [10]. In fact, these two species seem to share some morphological features with *Eochilotherium samium*, such as the flat to convex frontal region, the thickness of the nasal bones, and the highly elevated nuchal crest It seems most plausible that they do not belong to the genus *Chilotherium*; however, it is preferred not to include these species into the genus *Eochilotherium* at the moment. Nonetheless, until the issue about their generic attribution is resolved it is preferable to keep them in their original genus as ‘*C.*’ *wimani* and ‘*C.*’ *primigenium* [10,39].

*Chilotherium persiae* (Pohlig, 1885) [19]

### Type material

Pohlig (1885) [19] erected the species without fixing a holotype, providing any specimen numbers, photographs, or any information about the collection which housed the material he studied. He mentioned that the material he collected himself in Maragheh (Iran) was sent to Halle (Germany) [51]. This material is now housed in the palaeontological collection of the MLU. Pohlig (1885, 1886) [19,51] specifically mentioned that there were four adult and one juvenile skull that he assigned to this species. He did not specify whether these were collected by himself or if he saw them in some other collection. In the collections of the MLU only one adult and two juvenile skulls of *C. persiae* were able to be relocated. However, Pohlig (1886) [51] mentioned explicitly the fact that the collection of fossils in the area was continued by Theodor Strauss, as explained also by Rodler (1885) [77], who joined the excavation in Maragheh, collecting for the NHMW. The fact that Pohlig was well aware of these excavations and also specifically mentioned the material housed at the NHMW [51,78], along with the fact that several skulls were deposited in the collection of the NHMW until 1885, make it likely that Pohlig had seen at least some of these skulls and counted them into the, in total, five skulls he mentioned [51]. Until further information becomes available, however, the type material cannot be determined with certainty.

### Type locality

Upper Miocene deposits of Maragheh (Iran); exact locality unknown.

### Diagnosis

Medium-sized *Chilotherium* species with a weakly to moderately depressed frontal region, straight and relatively thick nasal bones, parietal crests that are moderately separated from each other (minimal distance between parietal crests up to 54 mm, n = 11) and a unique combination of dental characters: M3 with a somewhat quadrangular outline; long crochet; small crista sometimes present in the premolars that may close off the medifossette; very strong mesial and distal constriction of the protocone, which is lingually ﬂattened, resulting in a very long antecrochet that may close off the median valley; a strong mesial constriction of the hypocone; crista rarely present in molars but when present may close off the medifossette; and a weak, discontinuous lingual cingulum mainly present at the entrance of the median valley in the premolars.

### Referred material

An axis (MNHN.F.MAR3939), five scapulae (MNHN.F.MAR1431, MNHN.F.MAR1433, MNHN.F.MAR2899, MNHN.F.MAR3898, and MNHN.F.MAR3900), 11 humeri (MNHN.F.MAR3901, MNHN.F.MAR3903, MNHN.F.MAR3904, MNHN.F.MAR3905, NHMW-GEO-2020/0014/0115, NHMW-GEO-2020/0014/0122, NHMW-GEO-2020/0014/0123, NHMW-GEO-2020/0014/0124, NHMW-GEO-2020/0014/0125, NHMW-GEO-2020/0014/0148, and NHMW-GEO-2020/0014/0147), 17 radii (MNHN.F.MAR1434, MNHN.F.MAR3906, MNHN.F.MAR3908, MNHN.F.MAR3909, MNHN.F.MAR3911, MNHN.F.MAR3912, MNHN.F.MAR3913, MNHN.F.MAR3963, NHMW-GEO-2020/0014/0107, NHMW-GEO-2020/0014/0108, NHMW-GEO-2020/0014/0109, NHMW-GEO-2020/0014/0110, NHMW-GEO-2020/0014/0111, NHMW-GEO-2020/0014/0126, NHMW-GEO-2020/0014/0127, NHMW-GEO-2020/0014/0128, NHMW-GEO-2020/0014/0149), a scaphoid (MNHN.F.MAR1412), two semilunars (MNHN.F.MAR1401 and MNHN.F.MAR1411), two pyramidals (MNHN.F.MAR1405, MNHN.F.MAR1413), a trapezoid (MNHN.F.MAR1409), six unciforms (MNHN.F.MAR1400, MNHN.F.MAR1403, MNHN.F.MAR1406, MNHN.F.MAR1408, NHMW-GEO-2020/0014/0140, and NHMW-GEO-2020/0014/0155), four second metacarpals (MNHN.F.MAR1375, MNHN.F.MAR1388, NHMW-GEO-2020/0014/0106, and NHMW-GEO-2020/0014/0142), five third metacarpals (MNHN.F.MAR1377, MNHN.F.MAR1379, MNHN.F.MAR1383, MNHN.F.MAR1429, and MLU.GeoS.6242), six fourth metacarpals (MNHN.F.MAR1376, MNHN.F.MAR1387, MNHN.F.MAR1390, MNHN.F.MAR1386, MLU.GeoS.6241, and NHMW-GEO-2020/0014/0143), seven femora (MNHN.F.MAR1416, MNHN.F.MAR1432, MNHN.F.MAR3920, MNHN.F.MAR3921, NHMW-GEO-1911/0005/0273, NHMW-GEO-2020/0014/0114, and NHMW-GEO-2020/0014/0152), 11 patellae (MNHN.F.MAR1463, MNHN.F.MAR3922, MNHN.F.MAR3923, MNHN.F.MAR3924, MNHN.F.MAR3925, MNHN.F.MAR3926, MNHN.F.MAR3927, MNHN.F.MAR3928, MLU.GeoS.6245, NHMW-GEO-2020/0014/0118, and NHMW-GEO-2020/0014/0130), 10 tibiae (MNHN.F.MAR1435, MNHN.F.MAR3931, MNHN.F.MAR3932, MNHN.F.MAR3933, MNHN.F.MAR3990a, MNHN.F.MAR4131, MNHN.F.MAR4132, NHMW-GEO-2020/0014/0112, NHMW-GEO-2020/0014/0113, and NHMW-GEO-2020/0014/0117), 14 astragali (IPUW-MFN21652, MNHN.F.MAR1360, MNHN.F.MAR1366, MNHN.F.MAR1367, MNHN.F.MAR1368, MNHN.F.MAR1369, MNHN.F.MAR1370, MNHN.F.MAR1371, MNHN.F.MAR1372, MNHN.F.MAR1373, MNHN.F.MAR1419, NHMW-GEO-2020/0014/0102, NHMW-GEO-2020/0014/0129, and NHMW-GEO-2020/0014/0137), 19 calcanei (MNHN.F.MAR1421, MNHN.F.MAR1422, MNHN.F.MAR1423, MNHN.F.MAR1424, MNHN.F.MAR1425, MLU.GeoS.6243, MLU.GeoS.6244, NHMW-GEO-2020/0014/0103, NHMW-GEO-2020/0014/0104, NHMW-GEO-2020/0014/0105, NHMW-GEO-2020/0014/0131, NHMW-GEO-2020/0014/0132, NHMW-GEO-2020/0014/0133, NHMW-GEO-2020/0014/0134, NHMW-GEO-2020/0014/0138, NHMW-GEO-2020/0014/0139, NHMW-GEO-2020/0014/0145, NHMW-GEO-2020/0014/0146, and NHMW-GEO-2020/0014/0151,), six naviculars (MNHN.F.MAR1407, MNHN.F.MAR1415, MNHN.F.MAR1427a, NHMW-GEO-2020/0014/0136, NHMW-GEO-2020/0014/0145, and NHMW-GEO-2020/0014/0156), three cuboids (MNHN.F.MAR1414, MNHN.F.MAR1427b, and NHMW-GEO-2020/0014/0145), three ectocuneiforms (MNHN.F.MAR1399, MNHN.F.MAR1427c, and NHMW-GEO-2020/0014/0145), three second metatarsals (MNHN.F.MAR1378, MNHN.F.MAR1381, and MNHN.F.MAR1385), three third metatarsals (MNHN.F.MAR1382, NHMW-GEO-2020/0014/0150, and NHMW-GEO-2020/0014/0154), and four fourth metatarsals (MNHN.F.MAR1452, NHMW-GEO-2020/0014/0121, NHMW-GEO-2020/0014/0144, and NHMW-GEO-2020/0014/0154).

### Remarks

*Chilotherium persiae*, originally erected as *Rhinoceros persiae*, represents the first described rhinoceros species that was later included in the genus *Chilotherium* [7,51]. Maragheh, the type locality of the species, has brought to light a very diverse mammalian fauna based on a large amount of fossil bones [21,22,53,79]. Along with many skulls, the species *C. persiae* is represented also by a large number of postcranial elements housed in different institutions, including the MLU, MNHN, and NHMW. These fossils were excavated at different fossil sites in the area around Maragheh that correspond to different stratigraphical layers [23,e.g., 51,77]. More specifically the studied postcranial material comes from the sites Karajabad, Kara Kend, and Kopran that represent the middle and lower fossiliferous beds in Maragheh [21,55,56].

*Chilotherium habereri* (Schlosser, 1903) [24]

### Lectotype

Schlosser (1903) [24] assigned several teeth to the new species that he erected, *Rhinoceros habereri*, without assigning a holotype. Therefore, all studied teeth (Schlosser, 1903: plate 5, figs 5–10, 12–21; plate 7, figs 1–3, 6, 8, 10, 11) [24] constitute the syntype of *Chilotherium habereri*. The associated P3 and P4 (SNSB- BSPG 1900 XII 622), illustrated by Schlosser (1903, plate 5, fig 18) [24], are herein designated as the lectotype of the species under the provisions of ICZN Art. 74. These were also used as the basis for the identification of the species by Ringström (1924) [7], who revised its morphological affinities.

### Diagnosis

Medium- to large-sized *Chilotherium* species with a moderately depressed frontal region, straight nasal bones, parietal bones that are moderately separated and a unique combination of dental characters: long crochet; usually closed off, round medifossette; crista absent in molars; very strong mesial and distal constriction of the protocone, which is lingually ﬂattened, resulting in a long antecrochet that may close off the median valley at a very advanced wear stage; a strong mesial constriction of the hypocone of the molars; medifossette commonly closed in premolars; and a discontinuous lingual cingulum is mainly present in the premolars.

### Type locality

Upper Miocene red clay deposits in Shanxi (China); exact locality unknown.

### Referred material

A scapula (GPIT/MA/04974), four humeri (GPIT/MA/04831, GPIT/MA/04832, GPIT/MA/04833, and GPIT/MA/04834), three radii (GPIT/MA/04778, GPIT/MA/04786, and GPIT/MA/04791), a scaphoid (GPIT/MA/04784), two second metacarpals (GPIT/MA/04782 and GPIT/MA/04783), two third metacarpals (GPIT/MA/04756 and GPIT/MA/04782), a fourth metacarpal (GPIT/MA/04776) a fifth metacarpal (GPIT/MA/04756), a femur (GPIT/MA/04835), a patella (GPIT/MA/04781), a tibia (GPIT/MA/04836), three astragali (GPIT/MA/04763, GPIT/MA/04779, and GPIT/MA/04792), three calcanei (GPIT/MA/04792, GPIT/MA/04859, and GPIT/MA/04860), a navicular (GPIT/MA/04782), and a third metatarsal (GPIT/MA/04796).

### Remarks

*Chilotherium habereri* is the first chilothere species described from China [24]. It was originally erected as *Rhinoceros habereri* [24] and later assigned to the genus *Chilotherium* [7]. However, the type material comprises only isolated teeth and dental characters are evidently not reliable for species identifications in the genus *Chilotherium* (see for example [47]). Ringström (1924) [7] tried to address this issue by using a skull from a different, but probably closely situated, locality with identical teeth to the type material as a basis to redefine the species. However, the morphological features seen in these teeth that were used to define the species are subject to intraspecific variation within the genus *Chilotherium*, therefore assigning a skull to the same species based solely on the morphology of the premolars may prove difficult. This complicates the matter and shows that a detailed revision of the type material, along with the material studied by Ringström (1924) [7], is needed. For the purpose of the current study, we tentatively refer the chilothere material from Kutschwan housed in the GPIT to *C. habereri*, as also proposed in the initial description of the material [25,26] and later supported by Ringström (1924) [7].

Chilotherium schlosseri [Weber, 1905] [14]

### Neotype

A well-preserved skull (GPIH 3015) with an associated mandible (GPIH 3015a), designated by Kampouridis et al. (2023) [10].

### Diagnosis

A large *Chilotherium* species characterised by widely separated parietal crests (minimal distance between parietal crests always over 70 mm in adult individuals), notably depressed frontal and nasal bones, nasal bones that bear a central longitudinal groove on the dorsal side, and a unique combination of dental characters: very long crochet; very strong mesial and distal constriction of the protocone, which is lingually ﬂattened, resulting in a very long antecrochet that usually closes off the median valley at an early wear stage in all teeth; a prominent mesial constriction of the hypocone; crista frequently present that closes the medifossette; and a discontinuous lingual cingulum that is occasionally moderately developed in the premolars; often a closed prefossette is present in the P2; in addition to sporadically present enamel plications in the upper teeth; and discontinuous lingual and buccal cingulids in the lower teeth (after [10]).

### Type locality

Upper Miocene deposits of Samos Island (Greece); exact locality unknown.

### Referred material

An axis (GMM FO-14), two scapulae (GMM 563 and NHMW-GEO-1911/0005/0245), seven humeri (GMM 495, GMM 496, GMM 599, GMM 601, GMM 602, GMM FO-18, and GMM FO-24), two radii (GMM 561 and GMM 564), two scaphoids (NHMW-GEO-1911/0005/0152 and NHMW-GEO-2009z0089/0001), four femora (GMM FO-10, GMM FO-11, GMM FO-12, and NHMW-GEO-1911/0005/0273), 10 tibiae (AMNH 22818, GMM 497, GMM 594, GMM 595, GMM 596, GMM FO-13, GMM FO-19, GMM FO-27, NHMW-GEO-1911/0005/0497, and NHMW-GEO-1911/0005/0498), six astragali (AMNH Unnumbered, AMPG-SAM516, AMPG-SAM517, AMPG-SAM518, GMM 571, and NHMW-GEO-1911/0005/0424), three calcanei (AMNH-20794, AMNH-95122, and AMNH Unnumbered), a navicular (AMPG-SAM516), a cuboid (AMPG-SAM516), a second metatarsal (GMM 572), four third metatarsals (AMNH-22818, AMPG-SAM520, GMM 572, and NHMW-GEO-1911/0005/0220), and two fourth metatarsals (AMPG-SAM520 and GMM 572)

### Description and comparison

#### Axis.

**Fig 2:**
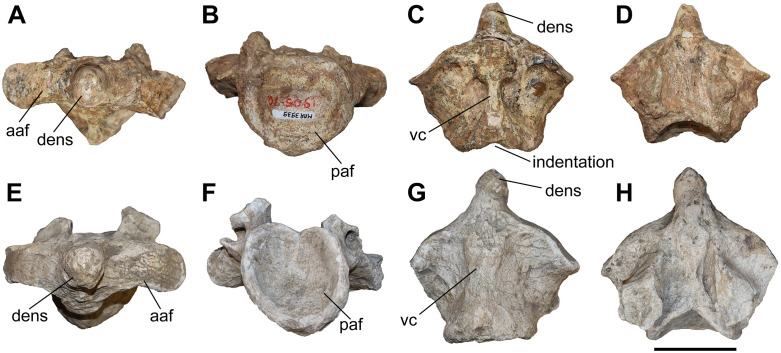
The axis of chilotheres. A–D, *Chilotherium persiae* (Pohlig, 1885) [19] (MNHN.F.MAR3939) from Maragheh (Iran) and E–H, *Chilotherium schlosseri* (Weber, 1905) [14] (GMM FO-14) from Samos (Greece) in anterior (A, E), posterior (B, F), ventral (C, G), and dorsal (D, H) views. Abbreviations: aaf, anterior articular facet; paf, posterior articular facet; and vc, ventral crest. Scale bar equals 5 cm.

The studied material includes only one axis belonging to *C. persiae* from Maragheh (MNHN.F.MAR3939) and one to *C. schlosseri* from Samos (GMM FO-14) (Fig 2). Both are missing the neural arch and preserve only the centrum of the vertebra. The two bones are very similar to each other, with the axis of *C. schlosseri* being slightly larger. The dens of the axis is quite narrow and elongated in comparison to the contemporary ‘*Ceratotherium*’ *neumayri*, in which the dens is much shorter, wider, and bears a prominent articular facet for the atlas on its ventral side (as seen in GMM FO-21, pers. obs.). In both *Chilotherium* species, the anterior articular facet of the axis is not as clearly defined, though this might be caused by some damage to the bone. The two vertebrae exhibit some slight differences like the ventral crest is wider and looks more massive in *C. schlosseri*, whereas in *C. persiae* it seems narrower and constricted in the middle. Concerning the posterior articular facet for third cervical vertebra, in *C. schlosseri* it has a high-oval shape with a slight, dorsal indentation, whereas in *C. persiae* it is more rounded, and the dorsal surface is flat. Additionally, in ventral view there is an indentation on the posterior side in *C. persiae* (Fig 2C), whereas *C. schlosseri* lacks such an indentation (Fig 2G). Lastly, the area where the lateral walls of the neural canal of the axis connect to the vertebral body seems to extend more posteriorly in *C. schlosseri* than in *C. persiae*. A single axis was described for *C. kowalevskii* from Grebeniki [15]. Unfortunately, the description and illustration provided for this axis [15] are not sufficient for a comparison to our specimens. Additionally, due to the small sample size it is not possible to assess the intraspecific variability. Nonetheless, it is very likely that at least some of the observed differences are of taxonomic value.

#### Scapula.

**Fig 3:**
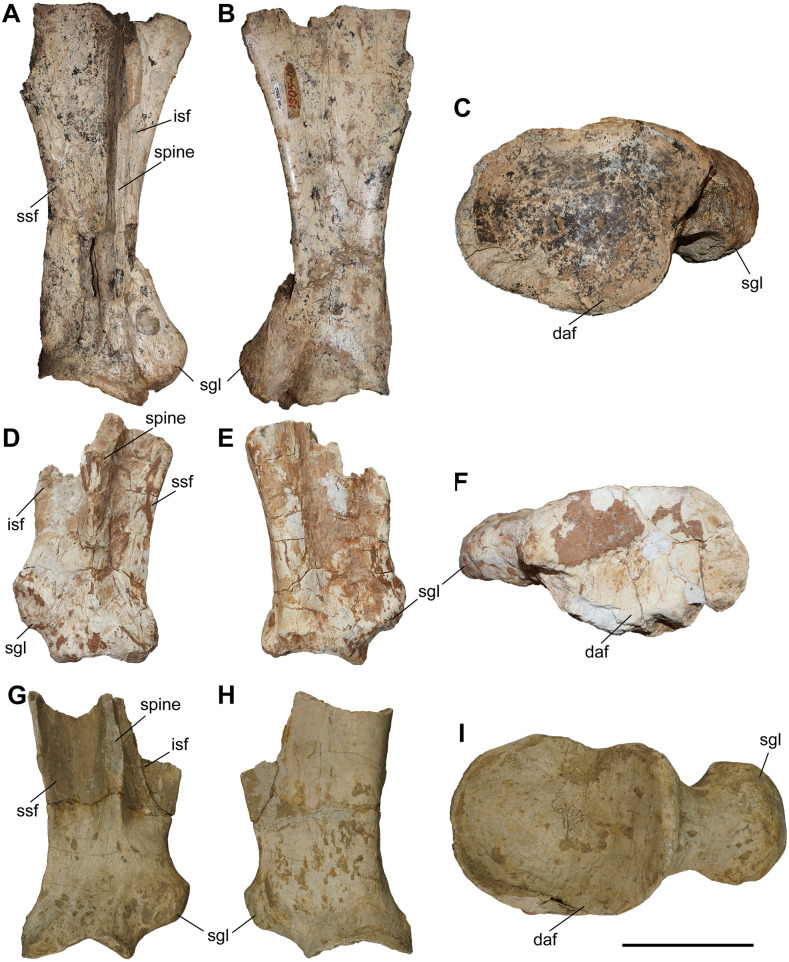
The scapula of chilotheres. A–C, *Chilotherium persiae* (Pohlig, 1885) [19] (MNHN.F.MAR3900, right) from Maragheh (Iran), D–F, *Chilotherium habereri* (Schlosser, 1903) [24] (GPIT/MA/04794, left) from Kutschwan (China), and G–I, *Chilotherium schlosseri* (Weber, 1905) [14] (GMM 428, right) from Samos (Greece) in anterior (A, D, and G), posterior (B, E, and H), and distal (C, F, and I) views. Abbreviations: daf, distal articular facet; isf, infraspinous fossa; sgl, supraglenoid tubercle; ssf, supraspinous fossa. Scale bar equals 10 cm for A, B, D, E, G, and H and 5 cm for C, F, and I.

The studied material includes five partial scapulae of *C. persiae* from Maragheh, one of *C. habereri* from Kutschwan, and two of *C. schlosseri* from Samos (Fig 3). Unfortunately, none of them are complete, but in all specimens the proximal portion is adequately preserved to compare them, and in some specimens parts of the scapular spine is preserved. The spine is well developed and straight. In the more complete specimens, it is visible that the infraspinous fossa is only slightly larger than the supraspinous fossa. A spinous tuberosity is present, but not very prominent. In all specimens the articular facet for the humerus is oval and anteroposteriorly elongated (as indicated by Guerin (1980) [31]). The articular facet does not seem to differ among the scapulae of *C. persiae*, *C. habereri*, *C. schlosseri*, and *C. wimani* [16] neither in size, nor in shape. The supraglenoid tubercle and the coracoid process form a continuous tuberosity, the shape of which varies to some extent in the studied specimens. It is more rounded in *C. persiae* and *C. schlosseri* (Fig 3C, I) and rather flattened in *C. habereri* (Fig 3F). Furthermore, is seems to be more protruding in *C. schlosseri*; however, the small sample size may obscure the variability of these features. The specimens of *C. persiae* do in fact exhibit some variety in their morphology.

### Humerus

**Fig 4:**
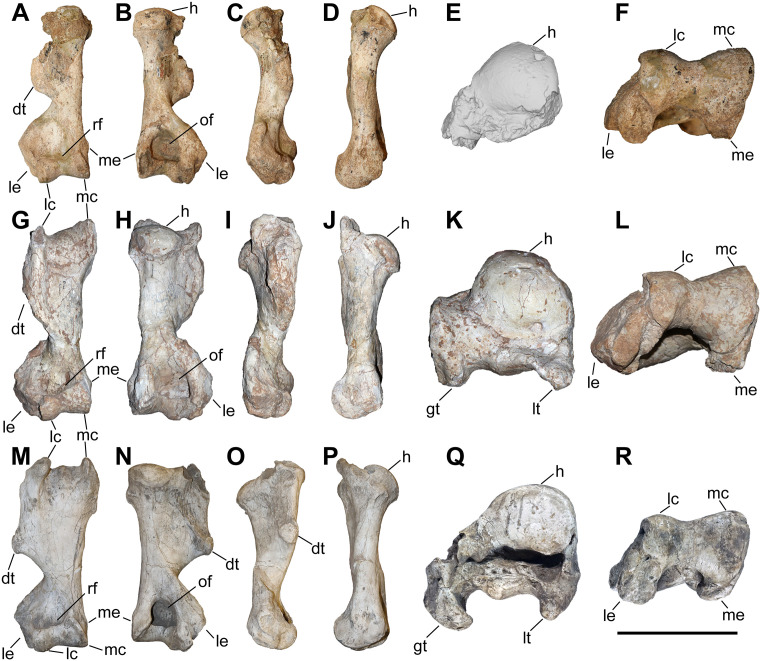
The humerus of chilotheres. A–F, *Chilotherium persiae* (Pohlig, 1885) [19] (MNHN.F.MAR3904, right) from Maragheh (Iran), G–L, *Chilotherium habereri* (Schlosser, 1903) [24] (GPIT/MA/04831, right) from Kutschwan (China), and M–R, *Chilotherium schlosseri* (Weber, 1905) [14] (GMM 601, right) from Samos (Greece) in anterior (A, G, and M), posterior (B, H, and N), medial (C, I, and O), lateral (D, J, and P), proximal (E: 3D model, K, and Q), and distal (F, L, and R) views. Abbreviations: dt, deltoid tuberosity; gt, greater trochanter; h, head of the humerus; lc, lateral condyle; le, lateral epicondyle; lt, lesser trochanter; mc, medial condyle; me, medial epidondyle; rf, radial fossa; and of, olecranon fossa. Scale bar equals 20 cm for A–D, G–J, and M–P, and 10 cm for E, F, K, L, Q, and R.

The studied material includes 12 humeri of *C. persiae* from Maragheh, four of *C. habereri* from Kutschwan, and seven of *C. schlosseri* from Samos (Fig 4, Table 1, S1 Table 1). Most specimens are only partially preserved. Their morphology is rather similar in all available specimens. The diaphysis is relatively straight. In the proximal part, the great and lesser tubercles are of similar size and their processes extend proximally to a similar degree, though the anterior part of the greater tubercle is broken in most specimens. The intermediate tubercle is somewhat less pronounced, without any distinct process. The two intertubercular grooves are smooth and relatively shallow. Anteriorly, the crest of the greater tubercle is prominent and extends distally to the deltoid tuberosity. The deltoid tuberosity is placed at the middle of the diaphysis. Its’ morphology and only be assessed in GMM 601 of *C. schlosseri* and GPIT/MA/04832 of *C. habereri*, where it is similarly well developed and posteriorly projecting. In all available specimens, on the posterior side also the tricipital line is well visible, represented by a ridge starting at the middle of the proximal part, approximately at the beginning of the greater tubercle and merged into the deltoid tuberosity, also marking the limit of the muscle attachment area there. In the distal part, the medial epicondyle is relatively weak, especially when compared to the prominent lateral one, with its crest reaching almost the middle of the shaft. The radial fossa is almost triangular and well-developed, placed above the trochlea of the humerus. The medial condyle of the trochlea is larger than the lateral one and they are not separated by a trochlear scar. The olecranon fossa is fairly rounded and deep in all specimens, but it seems to be somewhat narrower transversally in *C. schlosseri* (Fig 4N) than in *C. habereri* (Fig 4H). In the latter species, the opening of the olecranon fossa is much wider, and the fossa has overall a transversally slightly elongated oval shape, as seen in the four available specimens.

**Table 1:** Measurements (in mm) of the humeri of the studied chilotheres.

		L	Lcaput	TDprox max	TDprox art	APDprox	TDdia	APDdia	TDdist max	TDdist artic	APDdist	TDcaput	APDcaput
***C. persiae***	min		305.5				46.5	55.3	120	82.8	78.7	77.3	73.4
	max		351.4				63.9	63.4	139.5	93.4	94.4	78.1	75
	mean		327.2			98.1	55.9	58.4	131.7	87.8	84.4	77.7	74.2
	n		4			1	11	8	10	9	6	2	2
***C. habereri***	min	356	329	160	131.1	125.1	60	54.3	131.6	87.4	91.9	81.5	74
	max	365	334	161.3	138	127.2	63.4	62.3	150.1	98.4	97.4	94.6	90.1
	mean	360.5	331.5	160.7	134.6	126.2	61.8	57.8	142.7	93.9	95.5	88.4	84.4
	n	2	2	3	2	3	4	4	3	3	3	3	3
***C. schlosseri***	min	334	311	136	112.1	102	62.1	56.8	128.2	88.4	86	73	72.5
	max	353	320	152.2	134	139.5	76.2	78.6	146.8	98.3	91	84.1	83.8
	mean	343.5	314.3	145.5	126.6	124.7	69.1	63.0	138.0	93.9	89.5	79.9	78.4
	n	2	3	3	4	5	7	6	5	5	5	3	3

Unfortunately, for *C. persiae* it was only possible to measure the length from the caput humeri and not the full length of any specimen. For both *C. schlosseri* and *C. habereri* two complete humeri were studied respectively, allowing most dimensions to be measured (S1 Table 1).

The measurements provided for a well-preserved humerus of the type species *C. anderssoni* from its type locality Lok. 30 [7] shows that the humerus of *Chilotherium* is only little shortened compared to The comparison of the measurements of the herein studied chilotheres (Table 1, S1 Table 1), a well-preserved humerus of the type species *C. anderssoni* from its type locality Lok. 30 (China) [7], and several other chilothere humeri from the literature [11,12,16,17] to other rhinocerotids, like the aceratheriines *Aceratherium incisivum* Kaup, 1832 [80] from Höwenegg (Germany) [74] and *Acerorhinus zernowi* from Tung-gur (China) [75] shows that humerus of *Chilotherium* is only little shortened compared. Among chilotheres, ‘*C.*’ *wimani* from the Linxia Basin [16] and *E. samium* [14] seem to exhibit the smallest humeri, with the maximal length of the former being 335 mm and the single specimen of the latter being 312 mm. The species with the largest dimensions for the humerus seem to be *C. orlovi* from Pavlodar, with a length value range between 357 and 378 mm (n = 5) [12]. The dimensions of the humeri of *C. schlosseri*, *C. persiae*, *C. habereri*, *C. kowalevskii* from Grebeniki [15], and a potential chilothere from Loc. 51 of the Sinap Formation (Turkey) [81], are intermediate, with overlapping ranges.

### Radius

**Fig 5:**
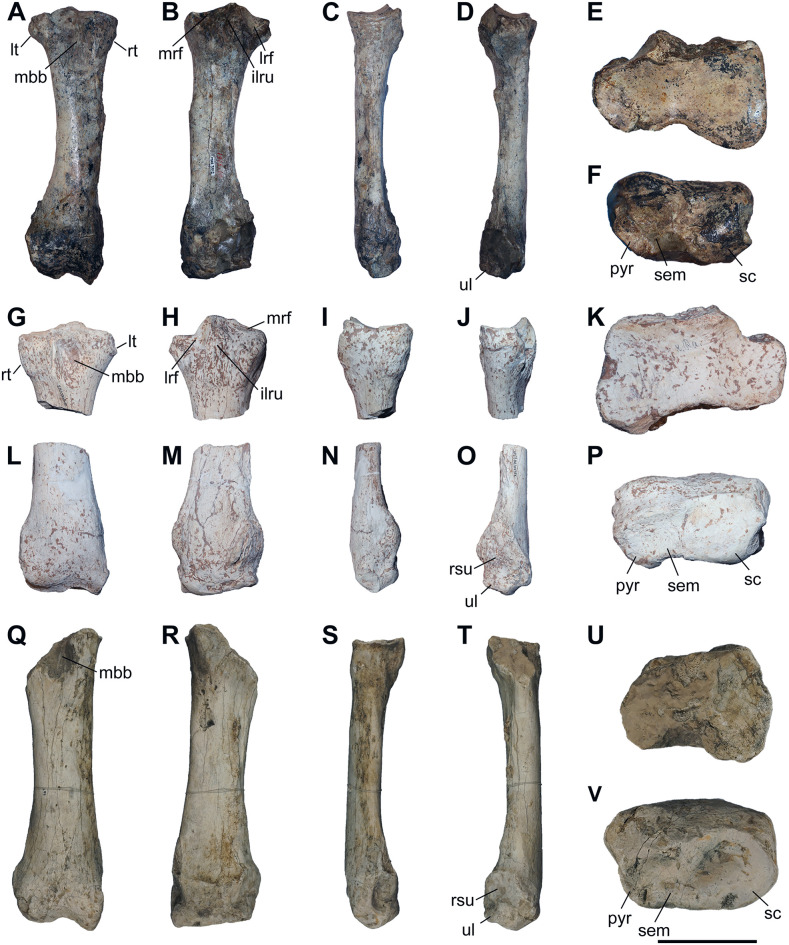
The radius of chilotheres. A–F, *Chilotherium persiae* (Pohlig, 1885) [19] (MNHN.F.MAR3912, right) from Maragheh (Iran), G–P, *Chilotherium habereri* (Schlosser, 1903) [24] (G–K: GPIT/MA/04878, left and L–P: GPIT/MA/04786, right) from Kutschwan (China), and Q–V, *Chilotherium schlosseri* (Weber, 1905) [14] (GMM 564, right) from Samos (Greece) in anterior (A, G, L, and Q), posterior (B, H, M, and R), medial (C, I, N, and S), lateral (D, J, O, and T), proximal (E, K, and U), and distal (F, P, and V) views. Abbreviations: ilru, attachment surface for the interosseous ligament between the radius and ulna; lrf, lateral radial facet; lt, lateral tuberosity; mbb, insertion for the musculus biceps brachii; mbb, medial radial facet; rt, radial tuberosity; pyr, articular facet for the pyramidal; rsu; rugose surface for the attachment of the ulna; sc, articular facet for the scaphoid; sem, articular facet for the semilunar; and ul, articular facet for the ulna. Scale bar equals 10 cm for A–D, G–J, L–O, and Q–T, and 5 cm for E, F, K, P, U, and V.

The studied material includes 17 radii *C. persiae* from Maragheh, three of *C. habereri* from Kutschwan, and two of *C. schlosseri* from Samos (Fig 5, Table 2, S1 Table 2). Most specimens are not complete and overall, poorly preserved. The proximal articular facet for the humerus is transversally elongated, subtrapezoidal in shape with the medial side being much wider anteroposteriorly than the lateral side. In the middle of the proximal facet, there is an indentation on the anterior side and a protrusion on the posterior side. In anterior view, below the proximal articular facet the insertion for the musculus biceps brachii is represented byan excavation in the middle of the bone, with a rugose surface. This insertion is much deeper in the only specimen of *C. habereri* where it could be assessed (Fig 5G) than in *C. persiae*, *C. schlosseri* (Fig 5A, Q), and *C. wimani* from the Linxia Basin [16]. However, in *C. persiae* it seems to cover a wider area. In GMM 564, the only specimen of *C. schlosseri* in which the proximal part of the radius is preserved, it is not complete, and the morphology of this feature cannot be studied in detail. Medially placed, next to the insertion for the m. biceps brachii, the radial tuberosity is visible. This is also much more prominent in *C. habereri* (Fig 5G), where it covers almost one third of the bone’s width in anterior view. In *C. persiae* (Fig 5A), the radial tuberosity is much smaller, and the surface is less rugose. In *C. schlosseri*, this feature cannot be observed. On the other side of the bone the lateral tuberosity is less prominent but extends towards the posterior side of the bone. On the posterior side, extending distally from the articular facet for the humerus, two articular facets for the ulna are visible. The medial one is a thin, transversally elongated stripe, whereas the lateral one is much larger and has an irregular shape and is larger in *C. persiae* than in *C. habereri* (Fig 5B, H). Around these two facets, a wide rugose area covers the whole proximal part of the posterior side of the bone, which represents an attachment area for the interosseous ligaments between the radius and the ulna. Further below a proximal interosseous space of about 2–3 cm is found in all specimens. From there on, the rugose crest for the attachment of the interosseous ligaments, runs down towards the distal part of the bone. A few centimeters above the distal epiphysis of the bone, a distal interosseous space of about 1 cm is placed. Right below this a large rugose area for the attachment of the ulna exists on the lateral side of the bone. At the distal end of this area, a small articular facet for the ulna is found, which seems somewhat larger in *C. persiae* and *C. schlosseri* (Fig 5D, T) than in *C. habereri* (Fig 5O). A discontinuous, rugose, and mediolaterally oriented crest is placed a few centimeters above the distal articulation. Its development varies between the specimens studied, even within the same species. For instance, for *C. persiae* in MNHN.F.MAR3908 it is rather weak, whereas in MNHN.F.MAR3906 it is much more distinct. In both specimens of *C. schlosseri* (GMM 561 and GMM 564) it is not very prominent. Concerning the two specimens of *C. habereri*, the crest is rather weak in GPIT/MA/04791 and almost absent in GPIT/MA/04786. Medially and laterally to this crest, two tuberosities are placed, which also exhibit a significant degree of variability. In *C. persiae* and *C. schlosseri* they have the shape of relative small tuberosities (Fig 5B–D, R–T), which may be somewhat proximodistally elongated in the case of MNHN.F.MAR3806 of *C. persiae*. In both specimens of *C. habereri* (Fig 5M–O), they actually form strong, proximodistally oriented crests of a few centimeters in length. The distal articular facets have a rather similar arrangement and form among the species. Medially, the articular facet for the scaphoid is large and anteroposteriorly convex. Laterally to the scaphoid facet the articular facet for the semilunar is smaller, subtrapezoidal to rounded in shape, and concave. Next to it the articular facet for the pyramidal is represented by a slightly obliquely placed, small stripe, which is not visible in all specimens. For instance, in MNHN.F.MAR3806 it is very faintly visible, whereas in MNHN.F.MAR3808 there is no trace of it, which may mean that it just is confluent with the facet for the semilunar.

**Table 2:** Measurements of radii (in mm) of studied chilotheres.

		L	TDproxmax	TDprox art	APDprox	TDdia	APDdia	TDdist max	TDdist artic	APDdist max	APDdist art
***C. persiae***	min	270.5	76.5	73.1	53.9	44.5	26.5	72.3	60.9	47.8	33.4
	max	295.2	90.6	89.4	63	55.5	35.3	90.5	77.3	55.9	39.8
	mean	280.1	85.2	82.8	56.5	49.5	31.9	81.2	71.0	52.0	37.1
	n	5	13	8	15	15	15	7	7	7	7
***C. habereri***	min		93.1	86.9	57.7	54	28.6	89.2	80.2	55	37.6
	max		93.1	86.9	57.7	58	33.8	95.1	86.8	60	40.6
	mean		93.1	86.9	57.7	56.0	31.2	92.2	83.5	57.5	39.1
	n		1	1	1	2	2	2	2	2	2
***C. schlosseri***	min						32.7	87.5	77.5	53.7	41
	max						33.3	92.9	77.8	54.1	43.4
	mean	290.0			55.0	55.7	33.0	90.2	77.7	53.9	42.2
	n	1			1	1	2	2	2	2	2

Concerning the dimensions of the radius, *C. sarmaticum* from Berislav exhibit the lowest values for the length of (247–280 mm) [11,16]. They are followed by those of ‘*C.*’ *wimani* from the Linxia Basin and *C. kowalevskii* from Grebeniki with value ranges for the length of 266–278 mm and 262–286 mm, respectively [11,17]. The radii of *C. orlovi* from Pavlodar exhibit the largest dimensions, with a value range of 292–320 mm for the length [12]. The only species in which the radii seem to reach or almost reach the lowest values of *C. orlovi*, are *C. schlosseri*, *C. persiae* (Table 2), and a potential chilothere from Loc. 12 of the Sinap Formation [81]. The single radius of *C. anderssoni* from Lok. 30, seems to show intermediate dimensions, with a length of 280 mm [7]. For *C. habereri* no complete radius is known, but the measurable dimensions indicate that the radii were quite large, and almost comparable to *C. orlovi*.

### Scaphoid

**Fig 6:**
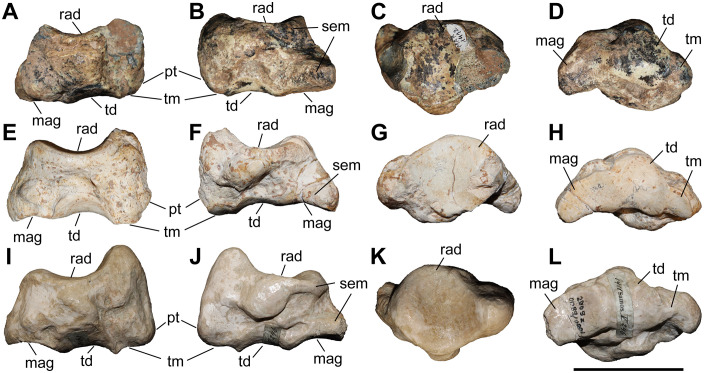
The scaphoid of chilotheres. A–D, *Chilotherium persiae* (Pohlig, 1885) [19] (MNHN.F.MAR1412, left) from Maragheh (Iran), E–H, *Chilotherium habereri* (Schlosser, 1903) [24] (GPIT/MA/04784, left) from Kutschwan (China), and I–L, *Chilotherium schlosseri* (Weber, 1905) [14] (NHMW-GEO-2009z0089/0001, left) from Samos (Greece) in anterior (A, E, and I), posterior (B, F, and J), proximal (C, G, and K), and distal (D, H, and L) views. Abbreviations: mag, articular facet for the magnum; pt, posterior tubersotiy; rad, articular facet for the radius; sem, articular facet for the semilunar; td, articular facet for the trapezoid; and tm, articular facet for the trapezium. Scale bar equals 5 cm.

The studied material includes one scaphoid of *C. persiae* from Maragheh, one of *C. habereri* from Kutschwan, and two from *C. schlosseri* from Samos (Fig 6, S1 Table 4). The specimens are fairly similar, though the Maragheh specimen is partially damaged. In proximal view, the articular facet for the radius is large with a subtrapezoidal outline and is in medial view sigmoidal. A prominent posteriolateral tuberosity is present, the morphology of which somewhat varies, medially to this a long thin stripe represents the proximal articular facet for the semilunar. In distal view, three articular facets for the trapezium, the trapezoid and the magnum are present. The medial facet for the trapezium is the smallest and its shape varies from triangular to semi-oval; this facet seems to be somewhat longer in *C. habereri* than in the other two species. The central facet for the trapezoid is the largest and has a subtrapezoidal shape with a convex medial border. The lateral articular facet for the magnum is subtriangular and connected to the laterodistal articular facet for the semilunar. The specimens are proximodistally short and lateromedially elongated, similar to ‘*C.*’ *wimani* from the Linxia Basin [16], *C. sarmaticum* from Berislav [11], and *C. orlovi* from Pavlodar [12]. Although, the measurements provided for the scaphoid of *C. orlovi* represent the largest values for any chilothere yet [12].

### Semilunar

A single partial semilunar (MNHN.F.MAR.1411) of *C. persiae* from Maragheh has been examined. Only the anterior portion of the bone is preserved. The proximal articular facet for the radius is saddle-shaped, with a posteriorly extending stripe and the anterior border of the facet being convex. In medial view, it bears two suboval articular facets for the scaphoid. In lateral view, two articular facets for the pyramidal are visible, the proximal facet is subtrapezoidal, whereas the distal one is a small longitudinal stripe. In distal view, only two articular facets are visible in the preserved part, the medioproximal one is the one for the scaphoid and the lateral facet articulates to the unciform and the preserves portion is subcircular. The articular facet for the magnum, which would have been placed next to these facets, is not preserved. The measurable dimensions of the Maragheh specimen are almost identical to the measurements provided for the semilunar of ‘*C.*’ *wimani* from the Linxia Basin [16]. Interestingly, they are significantly higher than the values provided for the dimensions of the semilunar of *C. sarmaticum* from Berislav and *C. orlovi* from Pavlodar [11,12]. It is possible that the measurements of the latter two species do not correspond to the same sections measured in *C. persiae* and ‘*C.*’ *wimani*, which follow those proposed by Guérin (1980) [31].

### Pyramidal

**Fig 7:**
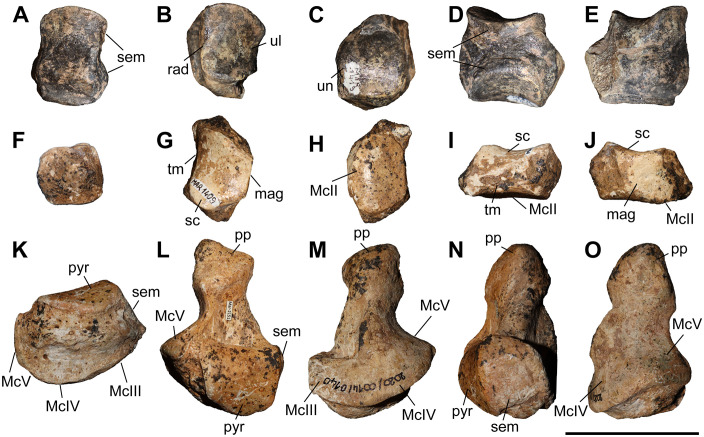
Carpals of *Chilotherium persiae* (Pohlig, 1885) [19] from Maragheh (Iran). A–E, pyramidal (MNHN.F.MAR1413, left), F–J, trapezoid (MNHN.F.MAR1409, right), and K–O, unciform (NHMW-GEO-2020/0014/0140, left) in anterior (A, F, and K), proximal (B, G, and L), distal (C, H, and M), medial (D, I, and N), and lateral (E, J, and O) views. Abbreviations: mag, articular facet for the magnum; McII, articular facet for the McII; McIII, articular facet for the McIII; McIV, articular facet for the McIV; McV, articular facet for the McV; pp, posterior process; pyr, articular facet for the pyramidal; rad, articular facet for the radius; sc, articular facet for the scaphoid; sem, articular facet for the semilunar; tm, articular facet for the trapezium; ul, articular facet for the ulna;and un, articular facet for the unciform. Scale bar equals 5 cm.

Two pyramidals (MNHN.F.MAR.1405 and MNHN.F.MAR.1413) of *C. persiae* from Maragheh have been examined (Fig 7A–E). They are higher than wide. The proximal facet, for the ulna, is antero-posteriorly concave and transversely slightly convex and indistinguishable from the probably much smaller facet for the radius. In medial view, two articular facets for the semilunar are visible, they are separated by a wide groove and have a semi-oval outline. In lateral view, in the distal half of the bone a well-developed tubercle exists that extends towards the posterior side of the bone. In posterior view, the articular facet for the pisiform has an obtuse subtriangular shape and contacts the articular facet for the ulna. The distal articular facet, for the unciform, is concave and has a suboval outline. The dimensions of the two pyramidals of *C. persiae* and those of ‘*C.*’ *wimani* from the Linxia Basin seem to overlap almost perfectly, despite the very limited sample size [16]. The single measurement for the pyramidal of *C. anderssoni* from Lok. 30 is also quite close to the height values provided for the other two species [7]. The values for the pyramidal of *C. orlovi* from Pavlodar are comparably higher [12]. The values provided for *C. sarmaticum* from Berislav [11] seem somewhat peculiar, as the given transversal diameter value is higher than the height of the bone, which is not the case in the other chilotheres and unusual for rhinoceroses in general. It would be more plausible to assume that distances measured by Korotkevitch (1970) [11], are not the same as those given by Guérin (1980) [31], which was followed in the current study.

### Trapezoid

Only one trapezoid (MNHN.F.MAR.1409) of *C. persiae* from Maragheh was studied (Fig 7F–J). In proximal view, the articular facet for the scaphoid has a subtrapezoidal outline. In medial view, it bears a small, elongated articular stripe for the trapezium. In lateral view, it bears a larger almost rectangular facet for the magnum. In distal view, the articular facet for the McII is oval. Deng (2002) [16] mentioned that the trapezoid of ‘*C.*’ *wimani* from the Linxia Basin in China bears two articular facets for the magnum on its lateral side, in contrast to the single articular facet present in the Maragheh specimen. The measurements also show that the Maragheh trapezoid is transversally wider (see S1 Table 7). The dimensions of the trapezoid of ‘*C.*’ *wimani* seem to be much closer to those of *C. sarmaticum* from Berislav [11]. The trapezoid of *C. anderssoni* from Lok. 30 seems to have dimensions that are more similar to *C. persiae* [7]. The trapezoid of the *C. orlovi* from Pavlodar, on the other hand, has generally greater dimensions, with only the transversal width being smaller [12]. It has to be noted that in complex bones like carpals, it is very difficult to compare measured dimensions if they did not follow the exact same method of measuring.

### Unciform

Six unciforms from Maragheh can be attributed to *C. persiae* (Fig 7K–O). In three of them the characteristic posterior process is broken off. In proximomedial view, the saddle-shaped facet for the pyramidal and the trapezoidal-shaped facet for the semilunar meet at an almost right angle. In distal view, the articular facets for the magnum, McIII, and McIV, are represented by a continuing wide stripe, without any well-defined separation. The articular facet for the magnum has posteriorly bent medial edge. The articular facet for the McIII has an irregular outline. The facet for the McIV is subtriangular to subtrapezoidal and forms a small, but notable, hump. In lateral view, the same articular stripe continues and forms the articular facet for the rudimentary McV. At the border between the articular facets for the McIV and McV a very subtle depression is present. In posterior view, a large curved posterior process is visible. The unciforms of *C. persiae* are much larger than those of ‘*C.*’ *wimani* from the Linxia Basin [16], especially the length of the bone in the latter species is significantly smaller. The dimensions reported for the unciform of *C. anderssoni* from Lok. 30 [7] and *C. sarmaticum* from Berislav [11] are only slightly smaller than those of *C. persiae*. The dimensions of the unciforms of *C. orlovi* from Pavlodar on the other hand are greater than in any other chilothere thus far [12].

### Second metacarpal

**Fig 8:**
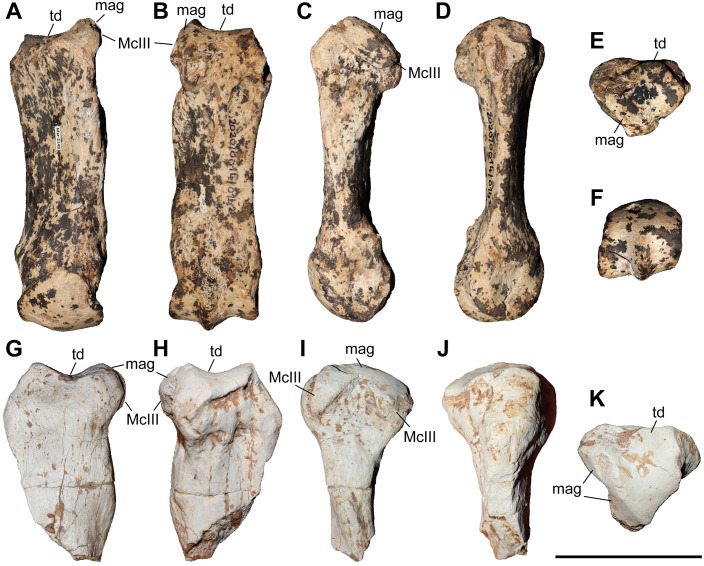
The second metacarpal of chilotheres. A–F, *Chilotherium persiae* (Pohlig, 1885) [19] (NHMW-GEO-2020/0014/0142, left) from Maragheh (Iran), and G–K, *Chilotherium habereri* (Schlosser, 1903) [24] (GPIT/MA/04783, left) from Kutschwan (China), in anterior (A, G), posterior (B, H), lateral (C, I), medial (D, J), proximal (E, K), and distal (F) views. Abbreviations: mag, articular facet for the magnum, McIII, articular facet for the McIII; and td, articular facet for the trapezoid. Scale bar equals 5 cm.

Four McII of *C. persiae* from Maragheh and two of *C. habereri* from Kutschwan were studied (Fig 8, Table 3). Only in a single specimen (NHMW-GEO-2020/0014/0142) the complete length of the bone is preserved and measurable, in the other specimens the distal portion is broken off. In proximal view the articular facet for the trapezoid is suboval to subtrapezoidal, transversally concave and anteroposteriorly convex. Laterally, it shares a wide connection to the obliquely placed longitudinal articular stripe for the magnum. The magnum facet contacts the articular facet(s) of the McIII in both species. In the two specimens of *C. persiae* where the proximal part is sufficiently preserved, the articular facet for the McIII is a continuous, wide stripe and is in proximal view concave (Fig 8C), whereas in the single specimen of *C. habereri* we see two small, distinct articular facets for the McIII (Fig 8I). No articular facet for the trapezium is visible on the proximomedial side of the bone. On the anterior side of the McII a prominent tuberosity covers the proximal part of the bone that represents a muscle attachment area for the extension of the metapodials. The tuberosity forms a slight discontinuous groove between the articular facet for the trapezoid and the tuberosity. On the posterior side, the articular facets for the trapezoid and magnum end on a slight protuberance, from which a small but notable crest extends distally to a variable degree. The shaft of the bone is straight, and the distal articular head is somewhat asymmetrical. The median distal keel develops in the posterior half of the trochlea. Deng (2002) [16] described a similar arrangement and overall morphology for the proximal articular facets of the McII mentioning also that the facets for the magnum and the McIII create a marked crest, by meeting at an angle. The length of the sole complete McII from Maragheh (Table 3, S1 Table 9) is slightly lower than the values given for *C. kowalevskii* from Grebeniki and *C. orlovi* from Pavlodar but is comparable to the length measured in *C. anderssoni* from Lok. 30, ‘*C.*’ *wimani* from the Linxia Basin, *C. sarmaticum* from Berislav, and a potential ‘primitive’ *Chilotherium* from Loc. 12 of the Sinap Formation [7,11,12,16,81]. The maximal transversal diameter of the proximal epiphysis is best comparable to those of ‘*C.*’ *wimani*, *C. kowalevskii*, *C. orlovi*, and the potential ‘primitive’ *Chilotherium* from Sinap, while the proximal anteroposterior diameter is closer to *C. sarmaticum* and *C. orlovi*.

**Table 3:** Measurements (in mm) of second metacarpals of the studied chilotheres.

		L	TDprox max	TDprox art	APDprox	TDdia	APDdia	TDdist max	TDdist artic	APDdist
*C. persiae*	min		37.1	31.1	36.3	30.4	15.4	34.6		
	max		42.5	39.6	41	33.1	15.7	37		
	mean	108.5	39.1	33.4	38.3	31.8	15.6	35.8	31.1	31.9
	n	1	4	4	4	2	2	2	1	1
*C. habereri*	GPIT/MA/04783		46.4	37	40					
	GPIT/MA/04782							40.3	37.5	33.3

### Third metacarpal

**Fig 9:**
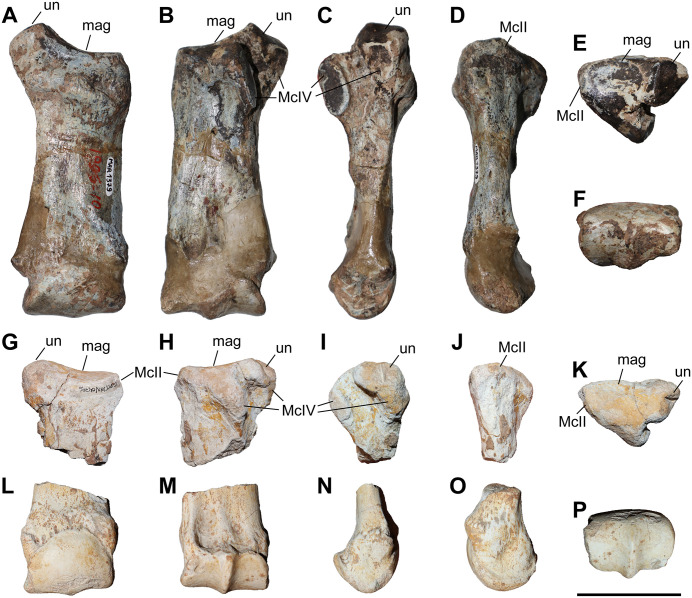
Third metacarpal of chilotheres. A–F, *Chilotherium persiae* (Pohlig, 1885) [19] (MNHN.F.MAR1379, right) from Maragheh (Iran), and G–P, *Chilotherium habereri* (Schlosser, 1903) [24] (G–K: GPIT/MA/04796 and L–P: GPIT/MA/04782, both right) from Kutschwan (China) in anterior (A, G, L), posterior (B, H, M), lateral (C, I, N), medial (D, J, O), proximal (E, K), and distal (F, P) views. Abbreviations: mag, articular facet for the magnum; McII, articular facet for the McII; McIV, articular facet for the McIV; and un, articular facet for the unciform. Scale bar equals 5 cm.

Five McIII of *C. persiae* from Maragheh and two of *C. habereri* from Kutschwan were studied (Fig 9, Table 4). In only two of those (MNHN.F.MAR.1379 and MNHN.F.MAR.1383) the complete length of the bone is measurable. The proximal articular facet for the magnum has an almost subtriangular to subtrapezoidal outline, with a sigmoidal anterior border. It is transversally concave and anteroposteriorly convex and meets the small, oval articular facet for the McIII at an almost right angle, on the medial side. Laterally in the anterior part, the facet for the magnum meets the trapezoidal articular facet for the unciform at an almost right angle. This facet, in turn contacts the semi-oval anterior articular facet for the McIV. This facet is separated by a wide and deep groove from the elliptical posterior articular facet for the McIV, which meets the posterior part of the articular facet for the magnum at an acute angle.

**Table 4:** Measurements (in mm) of third metacarpals of the *Chilotherium persiae* (Pohlig, 1885) [19].

		L	TDprox max	APDprox	TDdia	APDdia	TDdist max	TDdist artic	APDdist
*C. persiae*	min	132.9	45.9	39	24.4	17.1	46	46.5	35.6
	max	140.7	55.7	48.6	43.8	23.4	52.2	48.7	36.2
	mean	136.8	52.7	44.7	38.2	19.0	49.6	47.6	35.9
	n	2	4	4	4	5	3	3	2

All specimens have a rather straight shaft, with a slight concave lateral aspect in anterior view. In the proximal part of the anterior side, just below the proximal articular facet, a strong rugose surface for the insertion of the carpal extensor muscle exists. The distal articular head is fairly symmetrical, with a strongly convex proximal border on the anterior side and a prominent central keel on the posterior sides. The keel reaches approximately the middle of the head. Additionally, above this keel, on the posterior side, a small but prominent rugose crest extends proximally and vanishes before the middle of the bone. The overall morphology of the Maragheh specimens fits the descriptions provided for the McIII of other chilotheres like *C. anderssoni* from Lok. 30, ‘*C.*’ *wimani* from the Linxia Basin, *C. sarmaticum* from Berislav, *C. kowalevskii* from Grebeniki, and *C. orlovi* from Pavlodar [7,11,12,16]. Metrically, the main aspect that could be used to differentiate them seems to be the length of the bones (S1 Table 10). More specifically, the length of the Maragheh specimens (132.9 and 140.7 mm, Table 4) is comparable to the reported values for *C. kowalevskii* (128.9–142.7 mm, n = 10), *C. orlovi* (128–153 mm, n = 15) and the potential ‘primitive’ *Chilotherium* from Loc. 12 of the Sinap Formation (135 mm, n = 1), which are greater than the reported values for ‘*C.*’ *wimani* (120–123 mm, n = 2), *C. anderssoni* (127 mm, n = 1), and *C. sarmaticum* (113–129 mm, n = 6), having almost no overlap between them [7,11,12,16,81].

### APDAPD

#### Fourth metacarpal.

**Fig 10:**
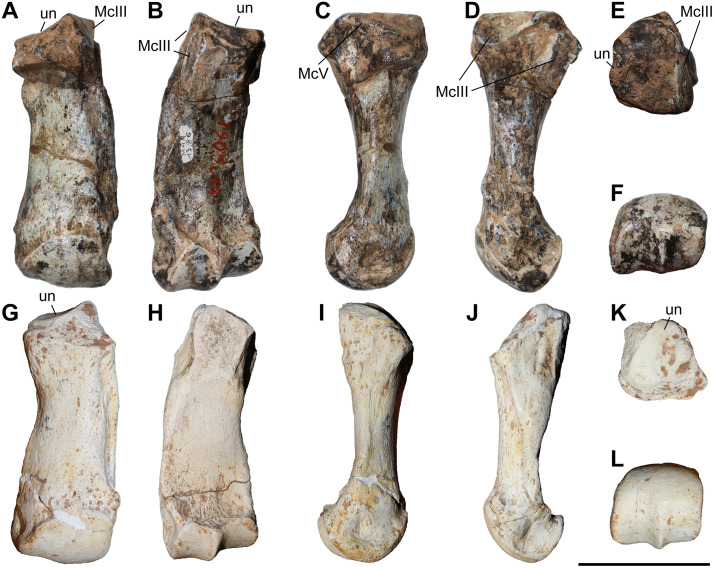
The fourth metacarpal of chilotheres. A–F, *Chilotherium persiae* (Pohlig, 1885) [19] (MNHN.F.MAR1386, right) from Maragheh (Iran), and G–L, *Chilotherium habereri* (Schlosser, 1903) [24] (GPIT/MA/04776, right) from Kutschwan (China), in anterior (A, G), posterior (B, H), lateral (C, I), medial (D, J), proximal (E, K), and distal (F, L) views. Abbreviations: McIII, articular facet for the McIII; McV, articular facet for the McV; and un, articular facet for the unciform. Scale bar equals 5 cm.

Six McIV of *C. persiae* from Maragheh and one of *C. habereri* from Kutschwan were studied (Fig 10, Table 5), all of which are more or less complete. In proximal view, the shape of the articular facet for the unciform varies from subtriangular to subtrapezoidal. Posteriorly it bends distally, creating additional articulation area for the unciform. In medial view, the two articular facets for the McIII are separated by a groove. The anterior articular facet for the McIII shares a wide contact to the articular facet for the unciform and form a ridge. Its’ shape varies from an elongated stripe to subtriangular. The posterior facet for the McIII either contacts the facet for the unciform only slightly or not at all. Its’ shape varies from almost circular to almost rectangular. On the lateral side, the articular facet for the rudimentary McV is a relatively thin and poorly-defined articular stripe that connects to the posterior extension of the unciform facet. Below the articular facets for the third metacarpal a long, almost triangular rugosity for the attachment of the interosseous ligament is placed. The shaft is slightly laterally curved, and the distal articular head is somewhat asymmetrical.

**Table 5:** Measurements (in mm) of fourth metacarpals of the studied chilotheres.

		L	TDprox max	TDprox art	APDprox	TDdia	APDdia	TDdist max	TDdist artic	APDdist
*C. persiae*	min	88.2	27.8	29.6	34.2	25.9	15.7	33.3	27.5	27.4
	max	106.1	35.8	29.6	43.1	34.2	20	39.6	36.4	34.6
	mean	101.1	30.9	29.6	40.1	30.6	17.9	37.1	32.3	31.7
	n	6	6	1	5	6	6	5	6	6
*C. habereri*	GPIT/MA/04776	100	>34			29.9	16.5	36.2	34.1	33.3

The McIV of the different chilotheres have a similar morphology overall. Though there are some potential differences found in the literature. For instance, while Korotkevich (1970) [11] and Bayshashov (1993) [12] described the existence of a small articular facet for the McV in *C. sarmaticum* from Berislav and *C. orlovi* from Pavlodar respectively, Deng (2002) [16] mentioned that in ‘*C.*’ *wimani* from the Linxia Basin there is no articular facet for the McV. However, no lateral view of any McIV is provided to confirm this and it is possible that the McV facet in ‘*C.*’ *wimani* remained unnoticed due to its very small size..

The metrical comparison of the McIV (S1 Table 11) indicates that there is a distinction between some species based on the values of the total length of this bone. More specifically, *C. persiae* (100.6–105.6 mm, n = 5) fits best among the greater values seen in *C. kowalevskii* (93.2–108.2 mm, n = 10), and is somewhat smaller than *C. orlovi* (107–115 mm, n = 8) [11,12]. Whereas ‘*C.*’ *wimani* (91.5–93 mm, n = 4), *C. sarmaticum* (90.5–95 mm, n = 6), *Chilotherium* indet. from Kavakdere in Turkey (89.4–92.5 mm, n = 2) have smaller values [11,16,81]. The single McIVs of *C. anderssoni* (98 mm) and of the potential ‘primitive’ *Chilotherium* from Sinap (97 mm) have an intermediate length [7,81]. Additionally, some material from Loc. 26 in the Sinap region referred to as ‘*Acerorhinus* sp. nov.’, includes a McIV with a length of 95.5 mm [81], which could also be associated with the smaller group, and probably belongs to a chilothere and not to *Acerorhinus*, as previously suggested [42]. Its’ gracility index (TDdia/L = 30.3%) falls well into the value range of the chilotheres (27.5–34.8%, n = 14) and the overall morphology of this bone also fits the morphology seen in *Chilotherium*. A McIV (MNHN.F.TRQ329) from Küçükçekmece (Turkey) that was assigned to *C. schlosseri* is, with a length of 112 mm, longer than most *Chilotherium* species, being in the range of *C. orlovi* but very slender (TDprox = 29 mm and TDdia = 25 mm). Its’ gracility index (TDdia/L = 22.3%) is lower than in any available chilothere (27.5–34.8%, n = 14) and even lower than in the non-chilothere *Aceratherium incisivum* from Höwenegg (24.3–27.4%, n = 4) but falls well into the range of *Acerorhinus zernowi* from Tung Gur (19.8–23.9%, n = 3), which has a significantly more elongated appendicular skeleton than any chilothere.

### Fifth metacarpal

**Fig 11:**
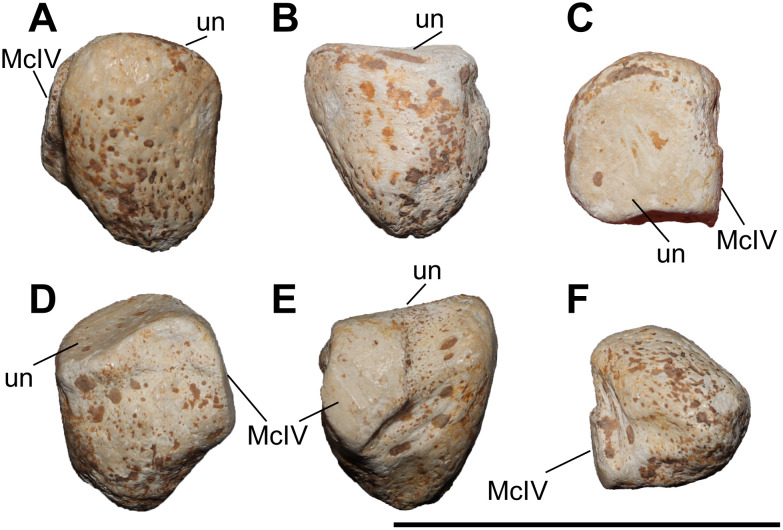
The fifth metacarpal of *Chilotherium habereri* (Schlosser, 1903) [24] (GPIT/MA/04756, left) from Kutschwan. A–F, McV in lateral (A), posterior (B), proximal (C), medial (D), anterior (E), and distal (F) views. Abbreviations: McV, articular facet for the McV; and un, articular facet for the unciform. Scale bar equals 5 cm.

A single McV (GPIT/MA/04756) of *C. habereri* from Kutschwan was studied (Fig 11). It is small, with a total length of 27.4 mm, and has a cone-like shape with a rounded tip. It has two articular facets: one large and quadrangular one for the unciform and a much smaller striper for the McIV. The two articular facets connect at a slightly acute angle and do not have any well-defined border. No other McV was found in the studied collections, and its morphology remains unknown for *C. persiae* and *C. schlosseri*. Ringström (1924) [7] described a simple, rudimentary McV for the type species *C. anderssoni* from Lok. 30. Deng (2002) [16] mentioned no McV of ‘*C.*’ *wimani* from the Linxia Basin and noted that in the studied McIV sample there is no articular facet for the McIV. Unfortunately, no lateral view of any McIV is provided, to confirm the complete lack of this facet. Korotkevich (1970) [11] and Bayshashov (1993) [12] explicitly mentioned the presence of a small articular facet for the McV in the McIV of *C. sarmaticum* from Berislav and *C. orlovi* from Pavlodar, respectively. Interestingly, the potential ‘primitive’ *Chilotherium* from Loc. 12 of the Sinap Formation exhibits a well-developed, and not at all rudimentary, McV [81], comparable to the McV of *Aceratherium incisivum* from Höwenegg [74]. This is in stark contrast to *C. habereri* and *C. anderssoni*, which have a McV that is similarly highly reduced to extant rhinoceroses. Hence, the ‘primitive’ *Chilotherium* from Loc. 12 of the Sinap Formation is likely to not represent at all a chilothere, but some other aceratheriine. Representatives of *Chilotherium* overall do not have a functional McV, confirming the notion of Ringström (1924) [7] that *Chilotherium* has tridactyl limbs.

### Femur

**Fig 12:**
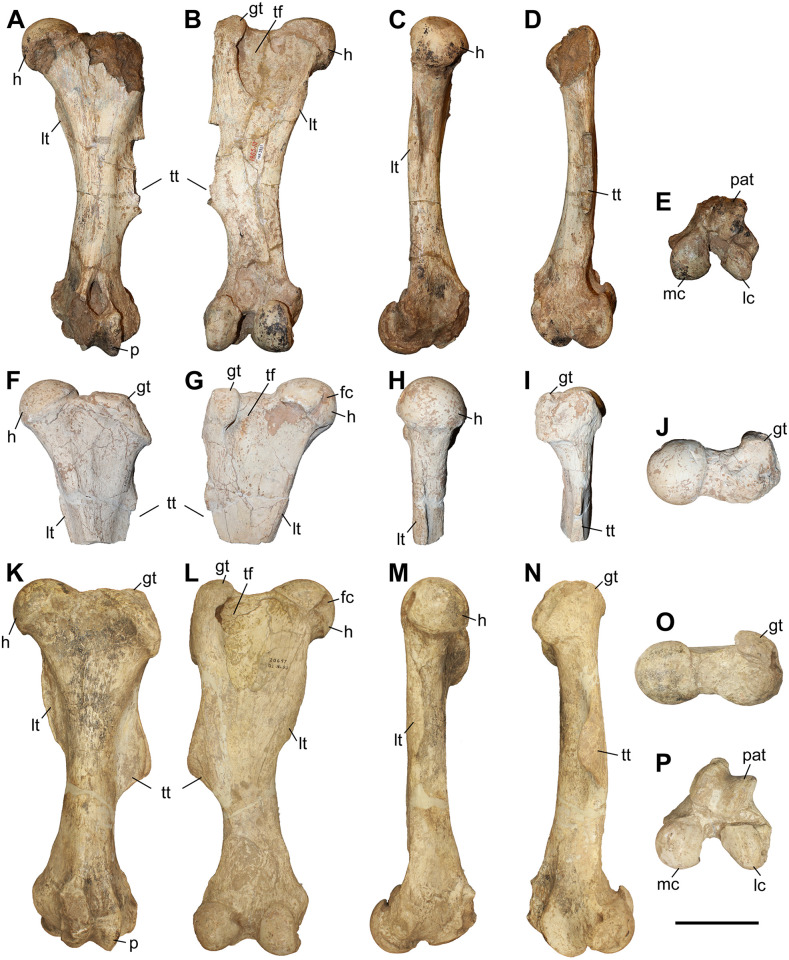
The femur of chilotheres. A–E, *Chilotherium persiae* (Pohlig, 1885) [19] (MNHN.F.MAR3921, left) from Maragheh (Iran), F–J, *Chilotherium habereri* (Schlosser, 1903) [24] (GPIT/MA/04835, left) from Kutschwan (China), and K–P, *Chilotherium schlosseri* (Weber, 1905) [14] (AMNH-20647, left) from Samos (Greece) in anterior (A, F, and K), posterior (B, G, and L), medial (C, H, and M), lateral (D, and N), proximal (J, and O), and distal (E, and P) views. Abbreviations: fc, fovea capitis, gt, greater trochanter; h, femoral head; mc, medial condyle; lc, lateral condyle; lt, lesser trochanter; pat, articular trochlea for the patella; tf, trochanteric fossa; and tt, third trochanter. Scale bar equals 10 cm.

Six femora of *C. persiae* from Maragheh, one of *C. habereri* from Kutschwan, and two of *C. schlosseri* from Samos were studied (Fig 12). Of these, only three, one of *C. persiae* and two of *C. schlosseri*, were complete enough to measure (almost) all dimensions of the bone. In most specimens the proximal part is either missing or damaged. The femoral head is protruding from the shaft and positioned higher than the greater trochanter, which is restricted to a slightly protruding, prominent tuberosity on the lateral side of the bone. On the posterior side of the femoral head, the fovea capitis is very high and narrow. On the lateral side, the convexity of the greater trochanter extends from the greater trochanter distally, creating the trochanteric fossa that is less prominent in specimen GPIT/MA/04835 of *C. habereri* (Fig 12G) than in the femora of *C. persiae* and *C. schlosseri* (Fig 12B, L). The lesser trochanter is represented by a small crest on the medial side of the bones, below the femoral head. The shaft narrows transversally towards the middle of the bone, with the third trochanter being moderately developed laterally and subtriangular when preserved, but is broken in almost all specimens. Distally, the trochlea for the patella is highly asymmetrical, with the medial lip extending much more proximally than the lateral one, in all specimens. On the anterior side, above the trochlea for the patella a deep fossa is visible (Fig 12A, K). On the posterior side, the two condyles for articulation to the tibia are slightly asymmetrical, with the medial one being wider and more rounded than the lateral one. They are separated by a deep intercondyloid fossa. The femur seems to be morphologically rather conservative within chilotheres, with most species looking almost identical [11,12,15,16]. Metrically, the femora of ‘*C.*’ *wimani* from the Linxia Basin [16] exhibits the smallest dimensions, with its length not surpassing 400 mm. On the other hand, the femora of *C. orlovi* from Pavlodar seem to exhibit the largest dimensions, with a length ranging from 438 mm to 485 mm [12]. The dimensions of *C. schlosseri* (S1 Table 12) and *C. kowalevskii* from Grebeniki seem to be somewhat intermediate but closer to *C. orlovi*, whereas the dimensions of *C. sarmaticum* from Berislav somewhat closer to those of ‘*C.*’ *wimani* [11,17].

### Patella

**Fig 13:**
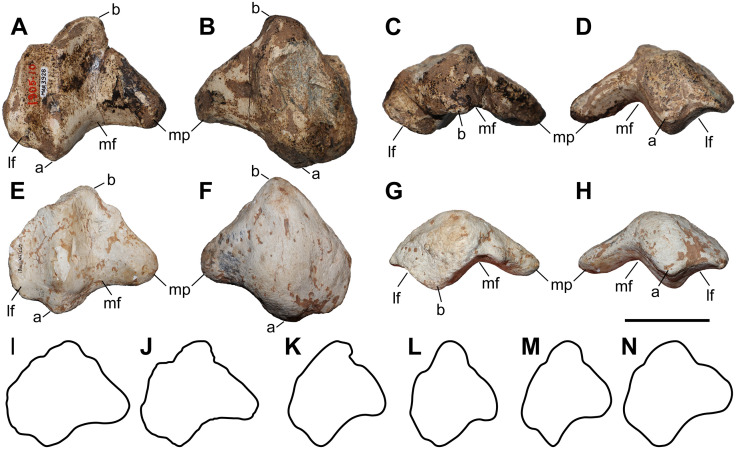
The patella of chilotheres. A–D, *Chilotherium persiae* (Pohlig, 1885) [19] (MNHN.F.MAR3928, left) from Maragheh (Iran), and E–H, *Chilotherium habereri* (Schlosser, 1903) [24] (GPIT/MA/04781, left) from Kutschwan (China), in anterior (A, E), posterior (B, F), proximal (C, G), and distal (D, H) views. I–N, outline of the patellae of *C. habereri* (I, drawn from ), *C. persiae* (J, drawn from Fig 13A), *C. kowalevskii* (K, drawn from [15]), *C. sarmaticum* (L, drawn from [11]), '*C*.' *wimani* (M, drawn from [16]), and *Aceratherium incisivum* (N, drawn from [74]) in anterior view. Abbreviations: a, apex; b, base; lf, lateral facet; mf, medial facet; and mp, medial process. Scale bar equals 5 cm for A–H. I–N are not in scale.

In total, 11 patellae of *C. persiae* from Maraghehand one of *C. habereri* from Kutschwan were studied (Fig 13, Table 6). The articular surface is asymmetrical, with the medial facet being higher than the lateral one, more so in *C. persiae* than in *C. habereri*. In *C. persiae*, the lateral side is narrow than in *C. habereri*. The base of the patella is rather high, projecting further proximally in *C. persiae* than in *C. habereri*. The apex is pointed in both species, but narrower in *C. persiae* and more rounded in *C. habereri* (compare Fig 13A, E). The medial process is rather prominent in both species but seems to be projecting slightly further in *C. persiae*. Metrically, the two species are very close (Table 6, S1 Table 13). The only other chilothere that has a patella with similar proportions is *C. orlovi* from Pavlodar [12]. The patella of ‘*C.*’ *wimani* from the Linxia Basin differs from the patellae of *C. persiae* and *C. habereri*, in being higher proximodistally [16]. More specifically, in ‘*C.*’ *wimani* the patella is higher than wide, which is not the case in *C. persiae* and *C. habereri*, which coincided with the proportions in *C. anderssoni* from Lok. 30 and *C. sarmaticum* from Berislav [7,11]. The measurements for a patella of *C. schlosseri* from Samos [14] are also close to these species and the proportions are a bit different from the patellae of *C. habereri* and *C. persiae* (S1 Table 13). The specimen itself was not figured [14] and was probably destroyed during WWII, along with the rest of the chilothere material from Samos that was housed in Munich [10,27,82]. For the patella of *C. kowalevskii* from Grebeniki no measurements are provided; based on the illustration provided by Pavlow (1913) [15], it seems to be very similar to that of ‘*C.*’ *wimani* and *C. sarmaticum* [16,45]. However, in *C. sarmaticum* the apex of the patella is much more rounded than in *C. kowalevskii* and ‘*C.*’ *wimani*. In the latter the tip of the apex is especially acute. The proportions of the patellae of *C. anderssoni*, ‘*C.*’ *wimani*, *C. kowalevskii*, and *C. sarmaticum* are closer to those seen in *Acerorhinus zernowi* from Tung-Gur and *Aceratherium incisivum* from Höwenegg [74,75]. The morphology of the two illustrated patellae of *Aceratherium incisivum* differ significantly from each other [74: Abb. 49C, 50]. However, it is mentioned that only one is complete and the outline of this specimen (Fig 13N) is intermediate between the very wide patellae of *C. habereri* and *C. persiae* and the much narrower ones of ‘*C.*’ *wimani*, *C. kowalevskii*, and *C. sarmaticum* (Fig 13I–M). Recently, Mallet and Houssaye (2024) [83] found that the shape of the patella in rhinoceroses and extant perissodactyls in general is strongly linked to their phylogenetic affinities and is less affected by functional constraints like body mass. Chilotheres seem to present an unexpected differentiation in the morphology of their patella and may be an exception to the otherwise rather conservative patella morphology among closely related taxa within Rhinocerotidae and other Perissodactyla.

**Table 6:** Measurements (in mm) of patellae of the studied chilotheres.

		L	TDprox max	TDprox art	APDprox
*C. persiae*	min	76.2	82		38
	max	89.8	95		54.4
	mean	84.65	89.5	80	48.6
	n	8	5	1	9
*C. habereri*	GPIT/MA/04781	83.4	90.7	76.2	45.6

### Tibia

**Fig 14:**
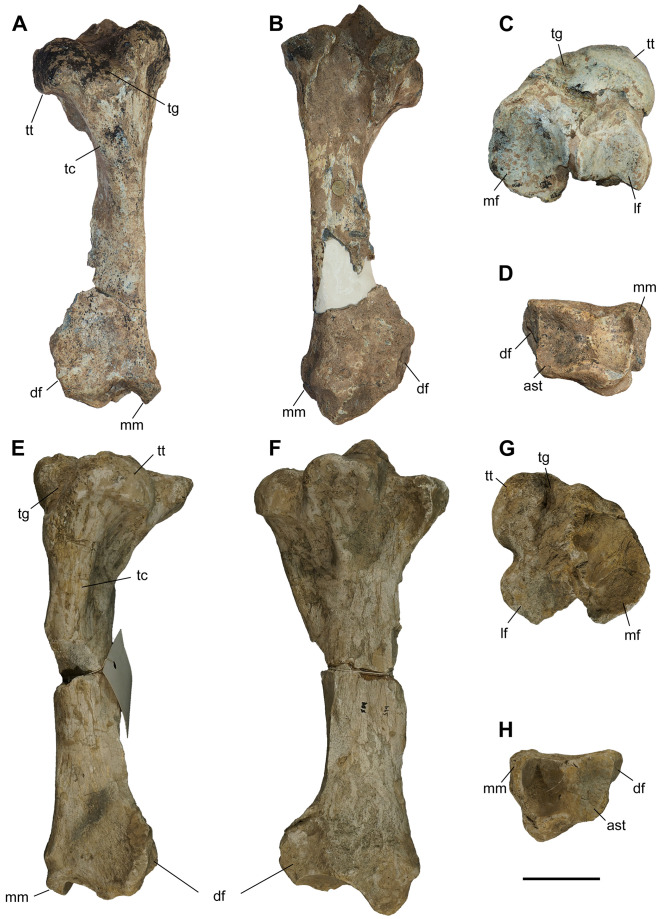
The tibia of chilotheres. A–D, *Chilotherium persiae* (Pohlig, 1885) [19] (A–B, D: MNHN.F.MAR3931 and C: MNHN.F.MAR3933, both right) from Maragheh (Iran), and E–H, *Chilotherium schlosseri* (Weber, 1905) [14] (GMM 594, left) from Samos (Greece) in anterior (A, E), posterior (B, F), proximal (C, G), and distal (D, H) views. Abbreviations: a, articular facet for the astragalus; df, distal articular facet for the fibula; lf, lateral articular facet for the femur; mf, medial articular facet for the femur; mm, medial malleolus; tc, tibial crest; tg, tuberosity groove; and tt, tibial tuberosity. Scale bar equals 5 cm.

Several tibiae of *C. persiae* from Maragheh, *C. habereri* from Kutschwan, and *C. schlosseri* from Samos have been studied (Fig 14, Table 7). However, only a few are adequately preserved to be able to measure the complete length of the bone and study their morphology in detail. They are rather similar with only a slight variability. The bone itself is relatively short, with the proximal epiphysis being quite wide. The lateral aspect of the tibia, on which the fibula is placed, is concave, whereas the medial side is straighter. In proximal view, the medial articular facet for the femur is larger than the lateral one. Both are concave and bend proximally towards the middle of the bone, creating a prominent ridge. Anterior to the articular facets for the femur a strong tibial tuberosityis placed, which functions as an attachment area for the patellar ligaments and merge distally into the tibial crest. The tibial tuberosity bears a wide and rounded tuberosity groove. The shape of the tibial tuberosity differs between *C. persiae* and *C. schlosseri* (compare Fig 13A, E). In proximal view, in *C. persiae* it is more transversally oriented, whereas in *C. schlosseri* it is anteroposteriorly oriented, being higher than wide. It is separated from the facet by a shallow groove. The central part of the diaphysis is relatively thin compared to the epiphyses, and relatively straight. On the lateral side of the distal epiphysis the rugose surface for the attachment of the fibula has a triangular outline. The articular surface for the astragalus has a subtrapezoidal outline in distal view, wider anteriorly than posteriorly, with the lateral malleolus of tibia and the posterolateral edge protruding.

**Table 7:** Measurements (in mm) of tibiae of the studied chilotheres.

		L	TDprox max	TDprox art	APDprox	TDdia	APDdia	TDdist max	TDdist artic	APDdist	APDdist art
*C. persiae*	min	270.7	98	95.8	87.1	35.3	38	63	58.8	55	40.2
	max	302.1	118.6	99.3	110.4	53.2	52.4	90.4	69.9	64.2	47.5
	mean	287.2	105.6	97.1	102.4	41.8	46.9	81.9	63.5	59.3	44.3
	n	7	5	3	6	7	6	8	7	8	6
*C. schlosseri*	min	282.9	115	102	76.8	32.6	44	78.4	57	62.3	41.3
	max	317	128.9	113.8	116.9	49.9	74.9	94.5	73.7	73.2	50.6
	mean	300.1	121.9	110.7	107.9	42.4	62.5	88.7	67.3	66.2	47.6
	n	2	6	6	6	9	8	5	5	5	5
*C. habereri*							50.9	87.3	71.5	61.6	53

The dimensions of the tibia are rather similar among chilotheres (Table 7, S1 Table 14), with the main differences being found in the total length and the dimensions of the proximal epiphysis [7,11,12,16]. *Chilotherium orlovi* from Pavlodar exhibits the greatest values for the length of the tibia, with rather large range of 280–340 mm (n = 11), which overlaps completely with the range for the length of the tibia of *C. kowalevskii* from Grebeniki (279–322 mm, n = 12). These value ranges, also cover the lengths measured in the tibiae of *C. schlosseri* from Samos (282.9–317 mm, n = 2), including also the tibia mentioned by Weber (1905) [14] from Samos with a length of 300 or 310 mm. The tibia mentioned by Kiernik (1913) [36] from Odessa has a lower length of 270/275 mm. The skull from the same locality can be assigned to *C. schlosseri*. The fact that the Odessa tibia is slightly smaller than the value range of *C. schlosseri*, is probably due to the small sample size (n = 2); especially when compared to the broad value range seen in *C. orlovi* and *C. kowalevskii*. The length of *C. persiae* tibiae from Maragheh is also very close to these values (270.7–302.1 mm, n = 7) and is almost identical to the value range given for *C. sarmaticum* from Berislav (271–301 mm, n = 11). The tibiae of ‘*C.*’ *wimani* from the Linxia Basin seem to be placed at the lowest end of this range or even below it (269–282 mm, n = 7). The only measured tibia of *C. anderssoni* from Lok. 30 (280 mm) and the only tibia of *Chilotherium* indet. from Kavakdere on which the length could be measured (276 mm) seem to exhibit a somewhat intermediate length, compared to the other chilotheres.

### Astragalus

**Fig 15:**
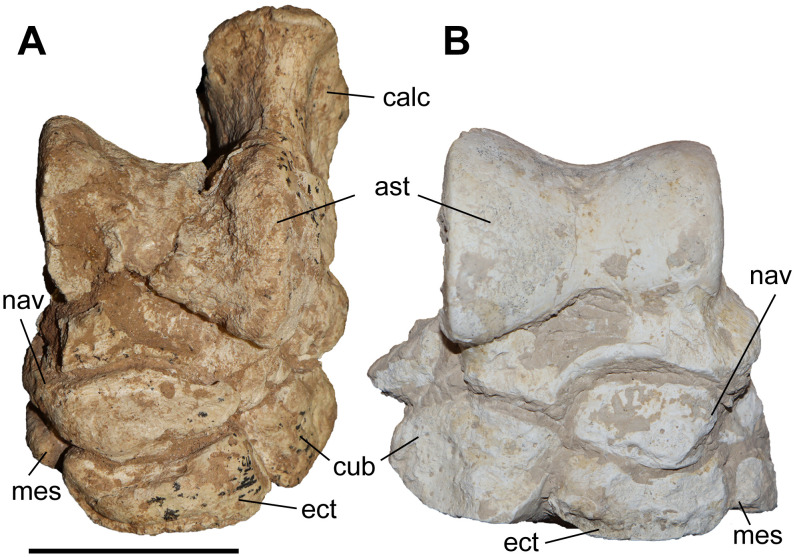
The tarsus of chilotheres. A, *Chilotherium persiae* (Pohlig, 1885) [19] (NHMW-GEO-2020/0014/0145, left) from Maragheh (Iran), and B, *Chilotherium schlosseri* (Weber, 1905) [14] (AMPG-SAM516, right) from Samos (Greece) in anterior view. Abbreviations: ast, astragalus; calc, calcaneum; cub, cuboid; ect, ectocuneiform; mes, mesocuneiform; and nav, navicular. Scale bars equal 5 cm.

**Fig 16:**
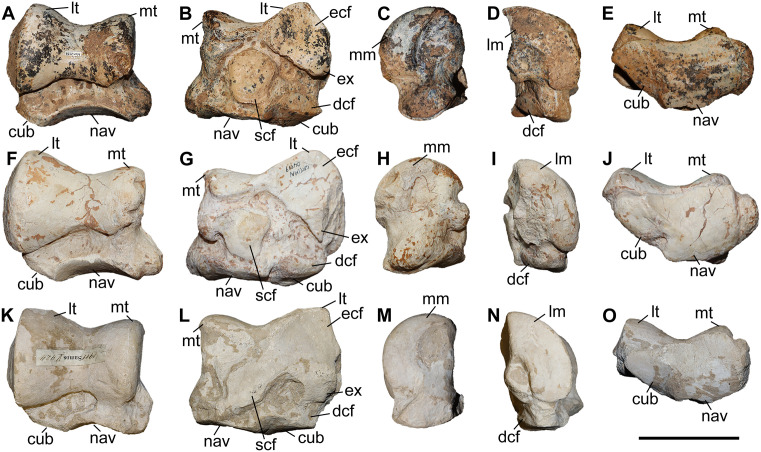
The astragalus of chilotheres. A–E, *Chilotherium persiae* (Pohlig, 1885) [19] (NHMW-GEO-2020/0014/01437, right) from Maragheh (Iran), F–J, *Chilotherium habereri* (Schlosser, 1903) [24] (GPIT/MA/04779, right) from Kutschwan (China), and K–O, *Chilotherium schlosseri* (Weber, 1905) [14] (NHMW-GEO-1911/0005/0424, right) from Samos (Greece) in anterior (A, F, and K), posterior (B, G, and L), medial (C, H, and M), lateral (D, I, and N), and distal (E, J, and O) views. Abbreviations: cub, articular facet for the cuboid; dcf, distal calcaneal facet; dpt; distally projecting tongue; ecf, ectal calcaneal facet; ex, distal expansion of the ectal calcaneal facet; lm, articular facet for thelateral malleolus of the fibula, lt, lateral lip of the trochlea, mm, articular facet for the medial malleolus of the tibia; mt; medial lip of the trochlea; nav, articular facet for the navicular; and scf, sustentacular calcaneal facet; Scale bar equals 5 cm.

**Fig 17:**
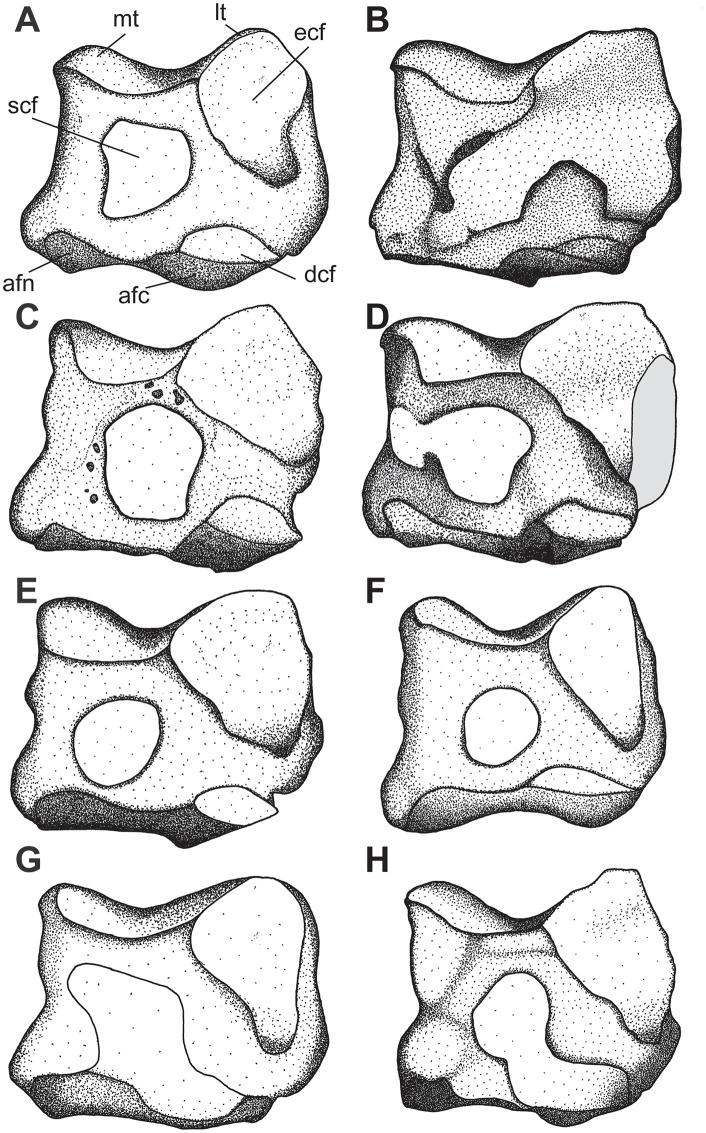
Schematic comparison of chilothere astragali in posterior view. A, *Aceratherium incisivum* Kaup, 1832 [80], B, *Chilotherium schlosseri* (Weber, 1905) [14], C, *Chilotherium persiae* (Pohlig, 1885) [19], D, *Chilotherium habereri* (Schlosser, 1903) [24], E, *Chilotherium orlovi* Bayshashov, 1982 [30], F, *Chilotherium kowalevskii* (Pavlow, 1913) [15], G, *Chilotherium sarmaticum* Korotkevitch, 1958 [45], and H, ‘*Chilotherium*’ *wimani* Ringström, 1924 [7]. Abbreviations: afc, articular facet for the cuboid; afn, articular facet for the navicular; dcf, distal calcaneal facet; ecf, ectal calcaneal facet; lt, lateral lip of the trochlea; mt, medial lip of the trochlea; and scf, sustentacular calcaneal facet. Not to scale.

Concerning the astragalus, 14 specimens of *C. persiae* from Maragheh, two of *C. habereri* from Kutschwan, and five of *C. schlosseri* from Samos were studied (Figs 15–17, Table 8). Most of the specimens are fairly well preserved, allowing detailed descriptions. In anterior view, the articular facet for the tibia is fairly asymmetrical, in all studied specimens. The lateral lip of the trochlea is slightly wider and higher than the medial one. In *C. habereri*, this asymmetry is much more pronounced than in *C. schlosseri*, where the lips of the trochlea are much more similar in size (Fig 16A, F, K). In medial view (Fig 16C, H, M), the articular stripe for the medial malleolus of the tibia has a concave distal border and it narrows proximally. In lateral view, the articular stripe for the lateral malleolus of the fibula is very wide in *C. persiae* and *C. schlosseri* (Fig 16D, N) and less so in *C. habereri* (Fig 16I), but in all three species it retains a constant width and establishes a wide connection to the ectal calcaneal facet. The contact between that lateral articular facet for the malleolus of the fibula and the trochlea forms a sharp, almost right angle in *C. schlosseri*, whereas in *C. persiae* and *C. habereri* this angle is more rounded and wider. In distal view, the articular facets for the navicular and the cuboid share a wide contact and form a slight ridge between them (Fig 16E, J, O). The former is very large, subtrapezoidal, anteroposteriorly convex, and transversally concave. The latter is a much thinner stripe, which contacts the distal calcaneal facet. In posterior view, the morphology of the calcaneal facets varies significantly between the different *Chilotherium* species (Figs 16, 17) and some variability is observed within the same species from a given locality [7,11,12,16]. The sustentacular facet is relatively large and its shape can vary from almost circular to high oval, and in some cases can have an irregular outline. In *C. habereri* and most studied specimens of *C. persiae* it remains separated from the articular facet of the navicular, cuboid and the distal calcaneal facet. However, in a few specimens a small contact with these facets can be established. In *C. schlosseri*, two astragali feature a completely isolated sustentacular facet, in two other specimens this portion cannot be studied, and in the last two astragali (GMM 571 and NHMW-GEO-1911/0005/0424) a unique condition is observed, where the sustentacular facet establishes a wide contact with the ectal calcaneal facet. This kind of connection is not observed in any other chilothere astragalus. The astragalus of ‘*C.*’ *wimani* from the Linxia Basin (Fig 17H) variably shows a connection between the sustentacular facet, the cuboid facet and the distal calcaneal facet [16]. In *C. sarmaticum* from Berislav (Fig 17G) the astragalus exhibits a similar connection between the articular facets, but it is much wider and may also involve the navicular facet; this pattern is observed in 10 of the 12 astragali of *C. sarmaticum* [11]. Additionally, the sustentacular facet is more rounded in ‘*C.*’ *wimani*, whereas in *C. sarmaticum* it is sub-trapezoidal (Fig 17H, G). The species *C. anderssoni*, *C. kowalevskii*, and *C. orlovi* exhibit the plesiomorphic arrangement of articular facets in rhinoceros’ astragali, in which the sustentacular facet is isolated [7,11,12], similar to *C. habereri* and most *C. persiae* astragali and as seen in the astragalus of *Aceratherium incisivum* from Höwenegg (Fig 17A) and *Acerorhinus zernowi* from Tung-Gur [75]. Concerning the ectal calcaneal facet, it has a similar subtriangular outline in most species (Fig 17). In lateral view it is only little concave, compared to other rhinocerotids like *Aceratherium incisivum* from Höwenegg [74], in which it has a more prominently sigmoidal profile in lateral view. In all chilotheres, the ectal facet has a distally projecting tongue, which is usually relatively small and subtriangular to suboval in shape. In *C. schlosseri*, however, this distal tongue has a much bigger surface than in the other species; in this case, *C. schlosseri* exhibits a more derived morphology.

**Table 8:** Measurements (in mm) of astragali of the studied chilotheres.

		H	DL	TD	TD maxi dist	APD art dist	TD art dist	APD inf
*C. persiae*	min	61.2	47	67.4	62.6	31.5	54	40.7
	max	67.3	54.3	85.2	80.1	39.4	70.8	47.2
	mean	64.5	49.9	78.8	72.7	35.8	64.6	44.0
	n	13	14	14	14	14	14	13
*C. habereri*	min	73.3	54	84.2		36.5		48
	max	76.3	60.4	94		43.7		54.4
	mean	74.8	57.4	89.1	79.1	40.1	65.4	50.9
	n	2	3	2	1	2	1	3
*C. schlosseri*	min	63.7	53.9	76.6	68	34.8	58	37.7
	max	70	58.3	91.4	73.2	41.4	70.6	50.1
	mean	66.0	55.0	81.3	70.5	39.1	64.7	46.0
	n	6	5	6	6	5	5	6

A last difference that can be observed in the astragalus of *C. schlosseri* in comparison to the other chilotheres is that in anterior view the medial lip is distally connected to the articular facet for the navicular, through a rugose bone growth which is observed in the six specimens where the area is observable (in the seventh this part is covered by sediment) In *C. persiae* and *C. habereri* the trochlea is separated by the distal articular facets for the navicular and the cuboid by a prominent groove, as is the case in most rhinoceroses.

Overall, it seems very difficult to differentiate between most of the *Chilotherium* species, solely based on the astragalus morphology. However, *C. schlosseri* seems to be the exception, because half of the available specimens exhibit a connection between the sustentacular and ectal calcaneal facets, which is not observed in the other chilotheres. Additionally, the same species also exhibits a larger distally projecting tongue on the ectal calcaneal facet and the medial lip of the trochlea is connected to the navicular facet, which is not the case in the other chilotheres. Thereby, *C. schlosseri*, could be separated from the other *Chilotherium* species, based on some features. The variability of these features is, however, not clear and therefore the identification of isolated specimens remains ambiguous. Interestingly, the species ‘*C.*’ *wimani* and *C. sarmaticum* feature a wide connection between the sustentacular and ectal calcaneal facets [11,16]. This is also observed in some specimens of *C. persiae*, but it is not clear whether this could be used as a diagnostic feature for some species or if it is a variable feature, which could potentially be observed in any given species, given a large enough sample.

### Calcaneum

**Fig 18:**
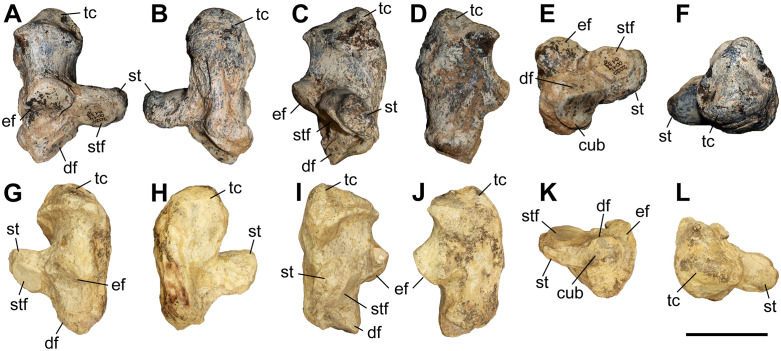
The calcaneum of chilotheres. A–F, *Chilotherium persiae* (Pohlig, 1885) [19] (NHMW-GEO-2020/0014/0139, right) from Maragheh (Iran), and G–L, *Chilotherium schlosseri* (Weber, 1905) [14] (AMNH-20794, left) from Samos (Greece) in anterior (A, G), posterior (B, H), lateral (C, I), medial (D, J), proximal (E, K), and distal (F, L) views. Abbreviations: cub, articular facet for the cuboid; df, distal articular facet, ef, ectal articular facet; st, sustentacular tali; stf, sustentacular articular facte; tc, tuber calcis. Scale bars equal 5 cm.

In total, 19 calcanei of *C. persiae* from Maragheh, three of *C. habereri* from Kutschwan, and three of *C. schlosseri* from Samos were studied (Figs 15, 18, Table 9). Most are well-preserved and allow a detailed comparison for this bone. The tuber calcis is well-formed in all species and has a rugose surface. On the posterior side of the bone, the surface from the tuber calcis to the distal articular facet for the cuboid exhibits a very rugose secondary bone growth in most bones. The sustentacular facet for the astragalus is rounded in most specimens, varying in shape from almost circular to subtriangular. It is isolated from the other articular facets in all studied specimen. The ectal facet has a generally subtrapezoidal outline and a sigmoidal profile in lateral view. It shares a wide contact with the semi-oval articular facet for the tibia in all three species. However, the tibia facet seems to be larger in *C. persiae* than in the other two species. The distal facet for the astragalus is elongated and subelliptical in most specimens. Distally it shares a wide contact with the articular facet for the cuboid, which is large and saddle-shaped.

**Table 9:** Measurements (in mm) of calcanei of the studied chilotheres.

		H	Hpost	APD sommet	TD sommet	TDmini post	APDbec	TDsust
*C. persiae*	min	89.8	58.8	46.8	35	30.5	55.4	64
	max	106.1	72.9	61.1	51.9	41.9	64.3	78.3
	mean	99.3	69.6	55.9	45.0	35.6	58.5	71.5
	n	13	19	18	17	18	14	8
*C. habereri*	GPIT/MA/04792	109.1	78	61	46	38	62.8	78.5
*C. schlosseri*	min	86.7		60	36.6	31.5	57.4	62.8
	max	107		63.2	55	48.5	64.1	80
	mean	96.9		61.8	45.7	38.8	60.1	71.5
	n	4		4	4	3	3	4

The morphology of the studied specimen does not allow a separation between the three species, due to the extreme similarity of the species and the intraspecific variability observed in the Maragheh sample. For instance, the shape of the tuber calcis may vary, in some specimens of *C. persiae* it is more posteriorly developed than in others. Based on the measurements found in the literature, it seems that *C. orlovi* from Pavlodar is larger [12], and more specifically has a greater height, than the other chilotheres. Whereas the calcanei of ‘*C.*’ *wimani* from the Linxia Basin [16] and *C. sarmaticum* from Berislav [11] are clearly smaller. On the other hand, the studied calcanei of *C. schlosseri*, *C. persiae*, and *C. habereri* (Table 9) overlap with the dimensions of *C. anderssoni* from Lok. 30 and *C. kowalevskii* from Grebeniki [11] and seem to have an intermediate size (S1 Table 17). Therefore, a specific identification based on the calcaneum is rather difficult.

### Navicular

Six naviculars of *C. persiae* from Maragheh, one of *C. habereri* from Kutschwan, and one of *C. schlosseri* from Samos were studied (Fig 15, 19A–B). One navicular of *C. persiae* (NHMW 2020/0014/0145) and the single specimen of *C. schlosseri* are found in an articulated tarsus and their morphology cannot be assessed (Fig 15B). In proximal view, the articular facet for the astragalus is subrhomboidal and anteroposteriorly concave. In distal view, the three articular facets for the cuneiforms are separated by weak ridges exist. The medial facet for the entocuneiform is the smallest, obliquely placed, and its shape varies from subcircular to almost triangular. The central facet for the mesocuneiform has a subtrapezoidal outline. The lateral facet for the ectocuneiform is the largest and its shape varies from subtrapezoidal to an upturned “L”. In lateral view, two articular facets for the cuboid exist. The proximal one is a narrow, anteroposteriorly oriented stripe, which is separated from the posterodistal facet by a shallow groove. The latter is obliquely placed and has an almost circular outline. The comparison of the studied material to chilotheres naviculars described in the literature shows that they are very similar. More specifically, Deng (2002) [16] described a very similar morphology for ‘*C.*’ *wimani* from the Linxia Basin and even mentioned the fact that it bears two separated articular facets for the cuboid. Comparing the dimensions given in the literature for ‘*C.*’ *wimani* [16] and *C. sarmaticum* from Berislav [11], it seems that *C. schlosseri* and *C. sarmaticum* have a similar height, which is lower than in *C. persiae* and ‘*C.*’ *wimani*. However, the overall dimensions of ‘*C.*’ *wimani* and *C. sarmaticum* are smaller [11,16] than in *C. schlosseri*, *C. persiae* and *C. habereri* (S1 Table 18). In the case of *C. orlovi* from Pavlodar [12] the measurements provided for the anteroposterior diameter of the navicular is greater than the transversal length, which is contrasting all other chilotheres and most rhinoceroses in general, therefore we assume that these values are in fact inversed. This would make the dimensions of *C. orlovi* fall into the size range of *C. schlosseri*, *C. persiae* and *C. habereri*, which is a much more plausible result.

**Fig 19:**
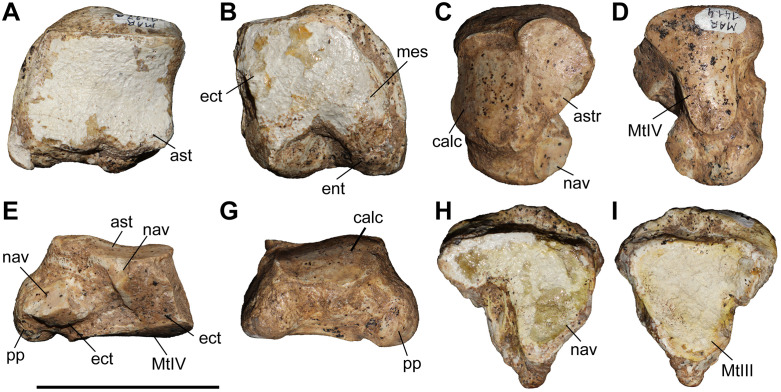
Tarsals of *Chilotherium persiae* (Pohlig, 1885) [19] from Maragheh (Iran). A–B, navicular (MNHN.F.MAR1427a, right) in proximal and distal views. C–G, cuboid (MNHN.F.MAR1414, left) in proximal, distal, medial, and lateral views. H–I, ectocuneiform (MNHN.F.MAR1424c, left) in proximal and distal views. Abbreviations: ast, articular facet for the astragalus; ect, articular facet for the ectocuneiform; ent, articular facet for the entocuneiform; mes, articular facet for the mesocuneiform; MtIII, articular facet for the MtIII; MtIV, articular facet for the MtIV; nav, articular facet for the navicular; and pp, posterior process. Scale bar equals 5 cm.

### Cuboid

Three cuboids of *C. persiae* from Maragheh and only one of *C. schlosseri* from Samos were available for study (Fig 19C–G). Of these, only two (MNHN.F.MAR1427b and MNHN.F.MAR1414) of *C. persiae* were able to be studied in detail, because the third specimen of *C. persiae* and the only one of *C. schlosseri* are articulated with other tarsal bones (Fig 15). In proximal view, two articular facets are separated by a slight ridge (Fig 19C). The medial facet for the astragalus has a subtriangular outline. The lateral facet for the calcaneum is subtrapezoidal. Together they form a subtrapezoidal surface with an irregular outline. In medial view, four articular facets are present that are connected in pairs, which are separated by a rugose groove (Fig 19E). In both pairs the proximal facet articulates to the navicular and the distal one to the ectocuneiform. The anterior facet for the navicular is a small triangle that contacts the proximal articular facet for the astragalus. The posterior one for the navicular is almost quadrangular and does not contact the facet for the astragalus. The anterior facet for the ectocuneiform is large, subrectangular, concave, and forms an obtuse angle with the anterior facet for the navicular, while also contacting the distal facet for the MtIV. The posterior facet is much smaller and almost quadrangular, forming an almost right angle with the posterior articular facet for the navicular and being separated from the MtIV facet. In distal view, the facet for the fourth metatarsal is large and almost triangular (Fig 19D). In the posterior part of the bone a large, rugose, and distally bent process is placed. The descriptions and illustrations provided for ‘*C.*’ *wimani* from the Linxia Basin [16] and for *C. orlovi* from Pavlodar [12] are generally similar to the morphology observed in the Maragheh specimens, but many details cannot be compared. In both species the articular facet for the astragalus is separated from the anterior facet for the navicular facet, as in *C. persiae*. In distal view, the anterior articular facet for the ectocuneiform is visible in *C. orlovi* [12], but is seems small and not concave, thereby possibly differing from *C. persiae*. The comparison of the metrical data for cuboids of *C. kowalevskii* from Grebeniki [17], ‘*C.*’ *wimani* [16], *C. sarmaticum* from Berislav [11], and *C. orlovi* [12] to the measured specimens of *C. schlosseri* and *C. persiae* (S1 Table 19) may offer some insight into the association of these specimen. More specifically, *C. persiae* has a relatively small cuboid with low length values (49.9 and 51.6 mm, n = 2) similar to *C. sarmaticum* (50.5 and 54.5 mm, n = 2) and ‘*C.*’ *wimani* (53–59 mm, n = 3). Whereas *C. schlosseri* (62 mm, n = 1) seems to be closer to the larger *C. kowalevskii* (60.8–66.2 mm, n = 6). The species *C. orlovi* exhibits a length range that overlaps with the other species (55–61 mm, n = 5). Overall, no clear separation of the species can be observed, and the specific identification would currently be impossible based solely on the morphometry of the cuboid.

### Ectocuneiform

Three ectocuneiforms of *C. persiae* from Maragheh and a single one of *C. schlosseri* from Samos were able to be studied. Only a single specimen of *C. persiae* is completely preserved (Fig 19H–I) and the single specimen of *C. schlosseri* is articulated with the other tarsal bones (Fig 15B). In proximal view, the articular facet for the navicular has an upturned “L” shape and is weakly concave. In medial view, in the proximal part an articular stripe for the mesocuneiform exists, while in the distal part two articular facets for the second metatarsal exist, which are separated by a prominent notch. On the lateral side the ectocuneiform articulates to the cuboid. The morphology of the articular facet(s) for the cuboid cannot be assessed due to the damage in this part of the bone. In distal view, the articular facet for the MtIII is generally triangular and resembles a mirrored, upturned “L”. The studied specimens fit the description for the ectocuneiform of ‘*C.*’ *wimani* (therein referred to an entocuneiform) from the Linxia Basin [16]. The ectocuneiform has a rather simple and uniform morphology and does not exhibit any diagnostic features. The metrical comparison of *C. kowalevskii* from Grebeniki [17], ‘*C.*’ *wimani* from the Linxia Basin [16], *C. sarmaticum* from Berislav [11], and *C. orlovi* from Pavlodar [12] to the measured specimens of *C. schlosseri* from Samos and *C. persiae* from Maragheh (S1 Table 20) shows that the dimensions of the ectocuneiform of most species overlaps even within the very small sample sizes. Therefore, any identification based on this bone seems impossible.

### Second metatarsal

**Fig 20:**
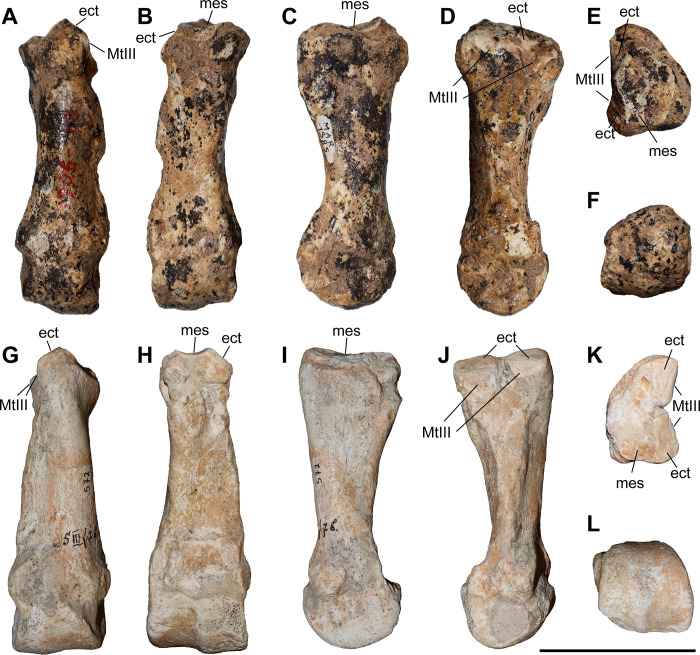
Second metatarsal of chilotheres. A–F, *Chilotherium persiae* (Pohlig, 1885) [19] (MNHN.F.MAR1385, left) from Maragheh (Iran), and G–L, *Chilotherium schlosseri* (Weber, 1905) [14] (GMM 572, right) from Samos (Greece) in anterior (A, G), posterior (B, H), medial (C, I), lateral (D, J), proximal (E, K), and distal (F, L) views. Abbreviations: ect, articular facet for the ectocuneiform; mes, articular facet for the mesocuneiform; MtIII, articular facet for the MtIII Scale bars equal 5 cm.

Three MtII of *C. persiae* from Maragheh and one of *C. schlosseri* from Samos were studied (Fig 20, Table 10). Most of them are more or less complete and allow a detailed description and metrical comparison. In proximal view, the articular facet for the mesocuneiform has a semioval outline and laterally contacts the articular facet(s) for the ectocuneiform, forming a slightly obtuse angle. Anterior to the mesocuneiform facet a rugose protrusion is present in the proximal part of the shaft, which extends towards the medial side and continuesdistally. There it connects to a slight tuberosity that is placed in the proximal part of the shaft. In lateral view, the MtII bears two separated articular facets in the only specimen of *C. schlosseri* (GMM 572) and two specimens of *C. persiae* (MNHN.F.MAR1378 and MNHN.F.MAR1381). In the third specimen of *C. persiae* (MNHN.F.MAR1385), a single continuous facet for the ectocuneiform exists. The facets for the ectocuneiform are connected to two separate, small articular facets for the MtIII. In *C. schlosseri*, the articular facets for the MtIII seem to be somewhat larger compared to those of *C. persiae*. In both species, the shaft has a subtriangular cross-section in the proximal part and is more oval and transversally elongated in the distal part; however, in *C. persiae* the cross-section is somewhat more rounded than in *C. schlosseri*. In medial view, on the proximal half of the shaft of *C. persiae* a well-developed, bulbous rugosity for the attachment of the interosseous ligament is placed (Fig 20A); in *C. schlosseri*, this rugosity covers a similar surface but is much more weakly developed (Fig 20G). On the anterior side of the bone, above the distal trochlea two bilateral protuberances are placed for the attachment of the ligaments of the fetlock joint. These are much more pronounced anteriorly in *C. schlosseri* than in *C. persiae*. The distal articular head is relatively rounded, asymmetrical with a convex proximal border on the anterior side. The sagittal keel of the articular head is rather weak and located mainly in the posterior part of the bone. On the posterior side, above the articular head two bilateral rugose protrusions are located.

**Table 10:** Measurements (in mm) of second metatarsals of the studied chilotheres.

		L	TDprox max	TDprox art	APDprox	TDdia	APDdia	TDdist max	TDdist artic	APDdist
*C. persiae*	min	93.4	28.6	18.5	35.8	20.8	17.4	27.5	26.7	29.8
	max	99	32.1	20.8	38.6	27.8	19.7	33.6	27.7	31
	mean	96.6	30.4	19.7	37.2	24.6	18.7	30.7	27.2	30.4
	n	3	2	2	3	3	3	3	2	2
*C. schlosseri*	min	102								
	max	102.4								
	mean	102.2	23.5	20.3	36.3	25.2	21.7	35.1	32.8	33.7
	n	2	1	1	1	1	1	1	1	1

The morphological differences observed in the MTII of the two species are rather slight. Metrically, however, there seems to be a distinction between larger and smaller species (Table 10, S1 Table 21). This is evident in species, where a larger sample size has been reported, like *C. kowalevskii* from Grebeniki, *C. sarmaticum* from Berislav, and *C. orlovi* from Pavlodar [11,12,17]. All three of these species, exhibit similar values for the transversal and the anteroposterior diameter of the diaphysis (TDdia and APDdia) of the MtII, but differ in some other dimensions of the bone and most prominently in the length. More specifically, *C. kowalevskii* and *C. orlovi* exhibit a higher value range for the length of the MtII (94.6–114.2 mm, n = 13 and 98–106 mm, n = 7 respectively) than *C. sarmaticum* (86.2–93 mm n = 6). Based on these well-sampled species, it is possible to associate *C. schlosseri* (102–102.4 mm, n = 2), *C. persiae* (93.4–97.3 mm, n = 3), *C. anderssoni* (97–99, n = 3), and a potential primitive *Chilotherium* (100.7 mm) with the larger species *C. kowalevskii* and *C. orlovi* [7,14,81]. Additionally, *C. schlosseri* from Odessa (95–110 mm) also falls into this size range. On the contrary, ‘*C.*’ *wimani* (89–94 mm, n = 3) and *Chilotherium* indet. from Kavakdere (85.2 mm) may be closer to the smaller *C. sarmaticum* [16,81]. It is rather surprising, that the MtII of the potential primitive *Chilotherium* from Loc. 72 of the Sinap Formation [81] has a length that is close to the length of species that are considered derived, such as *C. schlosseri*, *C. anderssoni*, and *C. orlovi* (S1 Table 21).

### Third metatarsal

**Fig 21:**
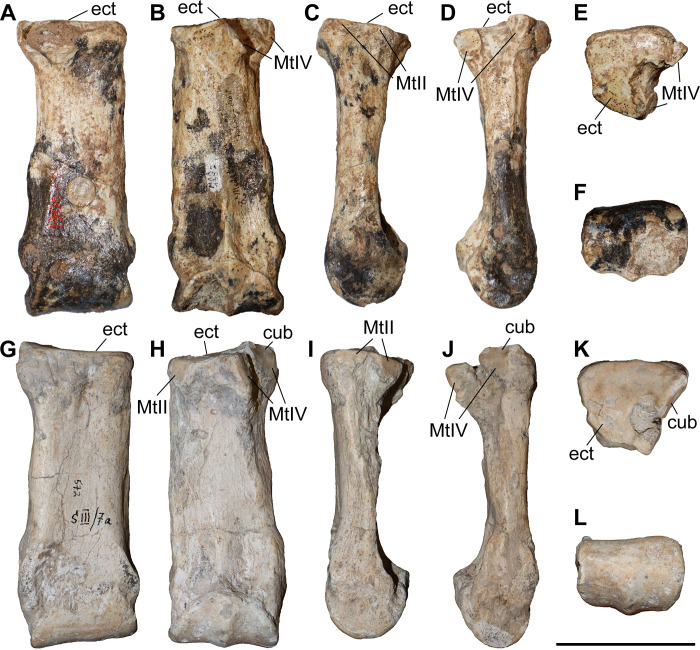
Third metatarsal of chilotheres. A–F, *Chilotherium persiae* (Pohlig, 1885) [19] (MNHN.F.MAR1385, right) from Maragheh (Iran), and G–L, *Chilotherium schlosseri* (Weber, 1905) [14] (GMM 572, right) from Samos (Greece) in anterior (A, G), posterior (B, H), medial (C, I), lateral (D, J), proximal (E, K), and distal (F, L) views. Abbreviations: cub, articular facet for the cuboid; ect, articular facet for the ectocuneiform; MtII, articular facet for the MtII; and MtIV, articular facet for the MtIV. Scale bars equal 5 cm.

Three MtIII of *C. persiae* from Maragheh and four specimens of *C. schlosseri* from Samos were studied (Fig 21, Table 11). Most of them are more or less complete and allow a detailed description and metrical comparison. Three specimens of *C. schlosseri* and one of the MtIII of *C. persiae* are completely preserved, except for some slight damage. The other specimens are lacking the distal half. In proximal view, the articular facet for the ectocuneiform covers the complete proximal surface of the bone and is almost triangular. The anterior border of the articular facet for the ectocuneiform is almost straight in *C. schlosseri* and has a very slight indentation in MNHN.F.MAR1382 of *C. persiae*. In medial view, two distinct facets for the MtII are visible in all specimens of both species; both facets are small and have a semi-oval outline. On the lateral side, three out of the four specimens of *C. schlosseri* feature an articular facet for the cuboid. In the fourth specimen (AMNH-22818) the relevant portion is somewhat damaged, and the potential presence of a cuboid facet is not clear. In the three specimens of *C. schlosseri* where it can be observed, the cuboid facet is small and trapezoidal, placed between the articular facet for the ectocuneiform and the anterior one for the MtIV (Fig 21G, J). In contrast to that, all three studied *C. persiae* specimens lack a cuboid facet (Fig 21B, D). In lateral view, two articular facets for the MtIV are placed below the ectocuneiform facet. These two facets are separated by a deep groove, which is much wider in MNHN.F.MAR1382 of *C. persiae*. In anterior view, the shaft widens distally towards the bilateral protuberances for the attachment of the ligaments of the fetlock joint. In posterior view, the sagittal keel of the articular head for the proximal phalanx is well developed. It placed in the posterior part of the articular head and in distal view, reaches the level of its medial rim in distal view.

**Table 11:** Measurements (in mm) of third metatarsals of the studied chilotheres.

		L	TDprox max	APDprox	TDdia	APDdia	TDdist max	TDdist artic	APDdist
*C. persiae*	min		39	34.1	32	18.2			
	max		40.4	37.7	35	20.1			
	mean	110.0	39.8	36.4	33.3	19.2	43.5	40.0	32.6
	n	1	3	3	3	2	1	1	1
*C. schlosseri*	min	111.2	41.3	33.8	36.7	18.2	46.2	42.4	30.3
	max	117	46.3	37	40	19.3	50	44.2	34.7
	mean	114.8	43.6	35.6	38.7	18.7	48.3	43.1	32.8
	n	4	4	3	4	3	3	3	3

The presence of a cuboid facet in MtIII may present a diagnostic feature separating *C. schlosseri* from at least some of the other species like *C. persiae* (Fig 21D, J). The type species *C. anderssoni* from Lok. 30 also lacks the cuboid facet [7]. Deng (2002) [16] mentioned that in both *C. anderssoni* and ‘*C.*’ *wimani* from the Linxia Basin (China) the MtIII does not bear an articular facet for the cuboid. Similarly, no cuboid facet has been described for *C. orlovi* from Pavlodar and in the illustrated MtIII, no such facet seems to be present between the articular facet for the ectocuneiform and that for the MtIV [12]. A MtIII from the Upper Miocene locality Küçükçekmece was assigned to *C. schlosseri* but was described as not having a cuboid facet. It is likely that this specimen, as well as the other *Chilotherium* specimens from Küçükçekmece actually belong to a different species and not *C. schlosseri*, which bears a cuboid facet. Unfortunately, for most chilotheres this condition has not been described or illustrated.

Metrically, however, there seems to be a slight distinction between larger and smaller species. More specifically, in *C. kowalevskii* from Grebeniki, *C. sarmaticum* from Berislav, and *C. orlovi* from Pavlodar the length of the MtIII seems to reach up to 120 mm, in the largest specimens [11,12,17]. The measured specimens of *C. schlosseri* (111.2–120 mm, n = 6) and *C. persiae* (110 mm) seem to have similar values to these species, with *C. schlosseri* having a mean value of about 116 mm, which is close to, but even higher than, the mean value of 113 mm seen in *C. kowalevskii* [11,17]. Respectively, *C. anderssoni* from China has a similar range for the length of the MtIII (110–118 mm, n = 3) as in these larger species [7]. The other Chinese species, ‘*C*.’ *wimani*, on the other hand exhibits a rather low value range for the length of the MtIII (100–109 mm, n = 3), not surpassing 110 mm. Although the sample size is rather small for most species – in many instances three specimen or less – it seems that ‘*C*.’ *wimani* differs somewhat from the other species, in having smaller dimensions. Similarly, the measured MtIII of a potential primitive *Chilotherium* from Loc. 72 of the Sinap Formation (106 mm) and of *Chilotherium* indet. from Kavakdere (101.5 mm) are below the value ranges of *C. kowalevskii* and *C. orlovi* or barely reaching the lowest values. It seems that the material from Turkey [81] has more similar values to ‘*C*.’ *wimani* and are also within the value range of *C. sarmaticum*.

### Fourth metatarsal

**Fig 22:**
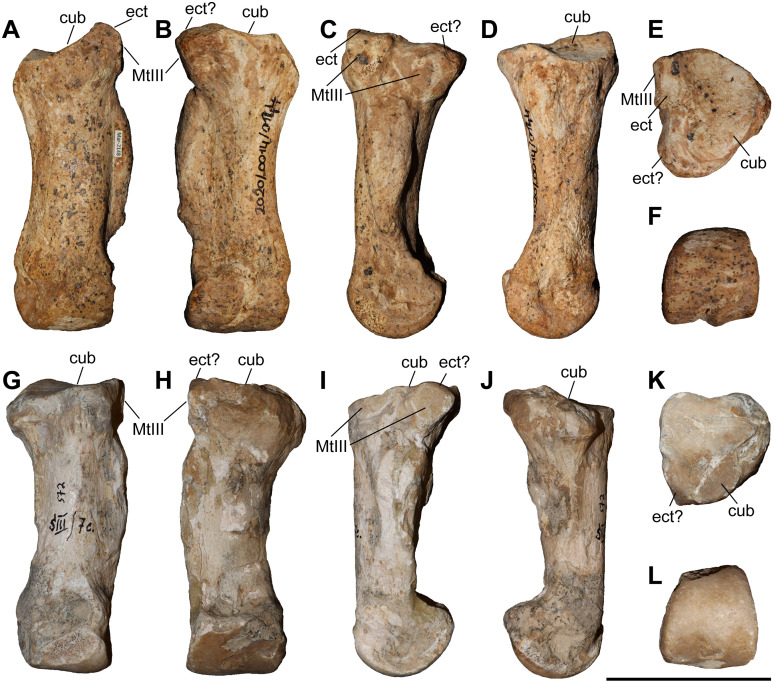
Fourth metatarsal of chilotheres. A–F, *Chilotherium persiae* (Pohlig, 1885) [19] (NHMW-GEO-2020/0014/0144, right) from Maragheh (Iran), and G–L, *Chilotherium schlosseri* (Weber, 1905) [14] (GMM 572, right) from Samos (Greece) in anterior (A, G), posterior (B, H), medial (C, I), lateral (D, J), proximal (E, K), and distal (F, L) views. Abbreviations: cub, articular facet for the cuboid; ect, articular facet for the ectocuneiform; and MtIII, articular facet for the MtIII. Scale bars equal 5 cm.

Four MtIV of *C. persiae* from Maragheh and two specimens of *C. schlosseri* from Samos have been studied (Fig 22, Table 12). Of these, only one specimen of each species (NHMW-GEO-2020/0014/0144 and GMM 572, respectively) is adequately preserved. The others are missing their distal parts in most cases. In proximal view, the articular facet for the cuboid is subtrapezoidal in GMM 572 of *C. schlosseri* and almost kidney shaped in NHMW-GEO-2020/0014/0144 of *C. persiae*. Below the articular facet for the cuboid, on the anterior side a small tubercle is present medially and a large one laterally, that covers the complete lateral side of the bone and weakens towards the posterior side. In medial view, the two articular facets that articulate to the MtIII vary in morphology between the two species. In *C. schlosseri* the anterior one is more elongated and thinner, while the posterior one is larger and rounded. In *C. persiae* the posterior one is similar to *C. schlosseri*, while the anterior one differs in being semi-oval and not elongated. Additionally, in *C. persiae* on the medial side, between the cuboid facet and the anterior MtIII facet, a small rectangular facet for the ectocuneiform is placed (Fig 22A, C, E). This differs from the morphology observed in *C. schlosseri*, where this facet is not found (Fig 22I). However, in both species the posterior MtIII facet seems to curve proximally, creating a very minute oblique surface, which could represent a ectocuneiform facet (Fig 22B, C, E, H, I, K), but cannot be confirmed, since there is no articulated set of tarsals and metatarsals. However, in both species the proximal portion of the posterior articular facet for the MtIII curves up, possibly creating a small articular surface for the ectocuneiform. Below the articular facets for the MtIII a prominent rugosity for the attachment of the interosseous ligament extends slightly beyond the middle of the shaft. This rugosity is more pronounced in *C. persiae* than in *C. schlosseri* (Fig 22A–C, G–I). The shaft of the bone is more or less straight. On the articular head for the proximal phalanx, there is only a very weak sagittal keel on the posterior side. In anterior view, the articular head is slightly asymmetrical.

**Table 12:** Measurements (in mm) of fourth metatarsals of the studied chilotheres.

		L	TDprox max	TDprox art	APDprox	TDdia	APDdia	TDdist max	TDdist artic	APDdist
*C. persiae*	min		35.5	30.3	32.3	29.2	18.2			
	max		43.3	31	41.3	37.8	27.2			
	mean	94.3	38.9	30.7	37.15	33.5	21.6		29.7	31
	n	1	4	3	4	2	3		1	1
*C. schlosseri*	min	91.7	33.4	39.4	35.1					
	max	96	35.5	39.4	40.9					
	mean	93.9	34.5	39.4	38	28	26.1	19.5	32.1	30.1
	n	2	2	1	2	1	1	1	1	1

The morphology of the articular facets for the tarsals and the MtIII may provide some insight into the separation of the species, as *C. persiae* and *C. schlosseri* exhibit significant differences. Most importantly, the existence of an ectocuneiform facet in the anterior part of the MtIV in *C. persiae* (Fig 22A, C, E) or the lack of it in *C. schlosseri* (Fig 22G, I, K) may present a diagnostic feature. Unfortunately, the presence of this facet cannot be assessed in the other chilotheres, as the descriptions and illustrations in the literature are not sufficient. However, this articulation is present in the non-chilothere aceratheriine *Aceratherium incisivum* from Höwenegg [74: Abb. 63]. No illustrations of the medial side of the MtIV is provided for *C. kowalevskii* from Grebeniki, but in anterior view it looks like such a articular facet for the ectocuneiform might be present [15,17]. The metrical comparison on the other hand seems to be more comparable (S1 Table 23). The species *C. kowalevskii* from Grebeniki and *C. orlovi* from Pavlodar have larger dimensions (length: 91.8–100.5 mm, n = 9 and 90–105 mm, n = 4, respectively) than *C. sarmaticum* from Berislav (length: 83.5–87 mm, n = 4), which do not overlap [11,12,17]. The length of the MtIV of *C. schlosseri* (91.7–96 mm, n = 4) and *C. persiae* (94.3 mm) fit much better the value ranges of the larger *C. kowalevskii* and *C. orlovi*. Whereas the dimensions of ‘*C*.’ *wimani* from the Linxia Basin (83–90 mm, n = 4), the potential primitive *Chilotherium* from the Sinap Formation (106 mm), and *Chilotherium* indet. from Kavakdere (83.4 mm), fit better those of the smaller *C. sarmaticum* [16]. Therefore, despite the small sample size in most species, a separation into two size classes can be observed, similar to observation by other authors [84].

## Discussion

Detailed comparisons of the cranial and dental material of different chilothere taxa [e.g., 8,10,49] suggest that their separation is a rather delicate topic. Nonetheless, based on certain features in the skull most species can be distinguished. For instance, it was recently shown that *Eochilotherium samium* differs significantly from most members of the genus *Chilotherium* and should be placed in its own genus within the Chilotheriina [10]. It was further established that two other species, ‘*C*.’ *wimani* and ‘*C*.’ *primigenium*, deviate from the typical *Chilotherium* morphology as exemplified by the type species *C. anderssoni* and probably also represent distinct genera [10,39]. Nonetheless, the tooth morphology of these species is remarkably consistent with *Chilotherium* and differs only in few features.

Within *Chilotherium* sensu stricto, the differentiation of species is less clear, as the skulls are very similar. The species *C. schlosseri* proves to be among the most recognisable species of the genus based on features such as the very prominent dorsally depressed skull and the very widely separated parietal crests (>70 mm, n = 12 [39]). Even the position of the nasal bones is affected by the frontal depression, and they are placed comparatively lower than in the other species, with the nasal bones being placed below the level of the dorsal border of the orbit in lateral view, in contrast to the other chilotheres [10,39]. However, there are characters that are able to also separate some of the other species. For instance, in the species *C. schlosseri* and *C. anderssoni* the skull is flattened, and the dorsal surface has a straight profile [10]. Whereas in species like *C. persiae*, *C. kowalevskii* and *C. sarmaticum* the skull is less flattened with the nuchal region being raised. Another morphological feature that could be used to separate some species is found in the frontal depression. More specifically, in some species like *C. persiae* and *C. kowalevskii* a faint longitudinal ridge is placed along the middle of the skull and is visible within the frontal depression [39]. This feature is not present in *C. schlosseri* and *C. anderssoni* [39]. Based on these features also a skull from the Upper Miocene locality of Reghiu (Romania), is morphologically closer to *C. kowalevskii* than to *C. schlosseri*. The two species were considered synonyms in the most recent re-evaluation of this skull, which led to the identification of the specimen as *C. schlosseri*, despite its closer affinities to *C. kowalevskii* [47]. This skull includes both a somewhat raised nuchal crest and a longitudinal ridge in the middle of the frontal depression like *C. kowalevskii*, also the parietal crests are 64.2 mm apart, thereby closer together than in *C. schlosseri* (>70 mm, n = 12), falling into the range of *C. kowalevskii* (40.1–66, n = 10) [10,17,39,47].

Unfortunately, in most cases within chilotheres the tooth morphology cannot solve the species determination. The lower teeth have an especially conservative morphology and are characterised by a notable uniformity within the group [8]. Despite that uniformity, the lower teeth can be used for the separation of chilotheres from other rhinoceroses, because in chilotheres the premolars are relatively shortened, resulting in a lower ratio of premolar to molar length [e.g., 42]. The morphology of the upper teeth is much more characteristic and can be used to separate chilotheres from all other rhinoceroses, but also includes some diagnostic features for the identification of some species within chilotheres. For instance, in the species *E. samium*, ‘*C*.’ *wimani* and ‘*C*.’ *primigenium* the premolars have either weakly constricted or unconstricted protocones. In the case of the P2, both the protocone and the hypocone are unconstricted and the median valley remains open even at a very late wear stage in these three species [10]. Also, the upper tooth morphology of *Shansirhinus* deviates from other chilotheres, exhibiting a prominent paracone fold and often a large number of enamel plications [73,76]. The sporadic occurrence of enamel plications is also observed in other chilotheres like *C. schlosseri*, where they are rather frequent, but not as abundant as in *Shansirhinus* [10].

Therefore, the taxonomy of the chilotheres has historically been very complicated and inconsistent but has started to be illuminated. In the present work we compare the postcranial material of chilothere species across Europe and Asia, in order to further elucidate the taxonomy of this group. Despite the relative abundance of cranial material from members of this group, the postcranial material is much more limited and rarely described in detail. This is probably due to a sampling bias towards skulls, which was especially common in the past. For instance, the *Chilotherium* material from the Upper Miocene of Kutschwan includes 11 cranial elements, but only 23 postcranial ones. Similarly, in the case of *C. schlosseri*, there are eight complete or almost complete skulls in different collections [39], but only very few collections house postcranial material of the species, namely the AMNH, AMPG, GMM, and NHMW.

**Fig 23:**
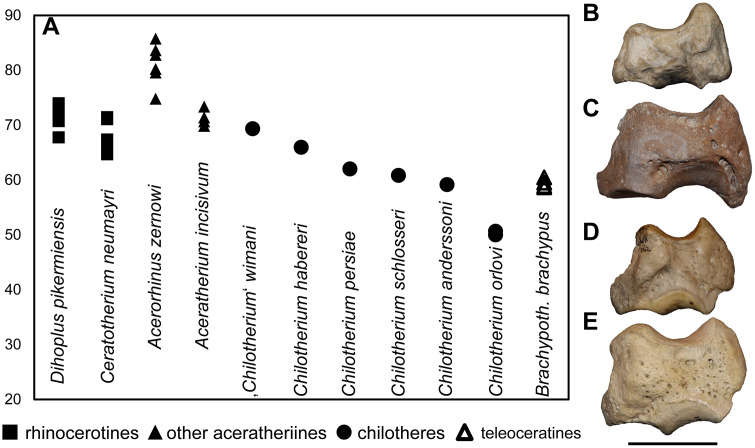
Comparison of chilothere scaphoids. A, univariate diagram of the height (H) to maximal length (L) ratio of the scaphoid of chilotheres compared to other rhinocerotids. B, left scaphoid of *Chilotherium schlosseri* (Weber, 1905) [14] (NHMW-GEO-2009z0089/0001) from the Upper Miocene of Samos (Greece); C, right scaphoid of *Brachypotherium brachypus* (GPIT/MA/03454, mirrored) from the Middle Miocene of Steinheim in Germany; D, left scaphoid of extant *Dicerorhinus sumatrensis* (NHMW-1500); and E, left scaphoid of extant *Ceratotherium simum* (NHMW-3086) in anterior view. Scale bar equals 5 cm. Measurements can be found in S1 Tables 4.

Nonetheless, when Ringström (1924) [7] coined the genus *Chilotherium*, he distinctly mentioned the rather peculiar anatomy of the appendicular skeleton that characterises this rhinocerotid group. He specifically noted that the extremities are strongly shortened, both the manus and pes are tridactyl with short metapodials, and that the abaxial metapodials are obliquely posteriorly oriented in comparison to the central one [7]. However, both in the work of Ringström (1924) [7] and the rest of the literature that was used in this context included rather short descriptions and comparisons of the postcranial elements of *Chilotherium* and their usefulness for taxonomic purposes was rather limited, as mainly the cranial and dental material was used to identify the species [e.g., 7,14,15]. However, the herein conducted comparison of the postcranial material of chilotheres confirms that there are several distinct features that characterise this genus, as already proposed by Ringström (1924) [7]. Most of these features are related to the shortening of the limbs. For instance, among the carpal bones the scaphoid is proximodistally notably shortened when compared to non-chilothere rhinocerotids for instance (Fig 23), with the only exception being teleoceratines like *Brachypotherium brachypus*. More specifically, most chilothere scaphoids have a height to length ratio between 50% and 69%, whereas in other Miocene rhinoceroses like *Dihoplus pikermiensis* and *Acerorhinus zernowi* this ratio can be higher, usually being above 70% and reaching up to 85% (Fig 23A). It has to be noted that this ratio is highly affected by the strongly proximodistally developed posterior tuberosity. In direct comparison it is clearly visible that the scaphoid of chilotheres is much shorter than that of extant horned rhinoceroses for instance (Fig 23B–E). More specifically, the anterior height is much lower than the posterior height in *Chilotherium*. This is a phylogenetically informative character also used in current morphological character matrices for rhinocerotids [34]. If only the anterior height was measured and used for the calculation, the ratio would be much lower. For *C. habereri* specifically instead of 66% it would be 53%, whereas in rhinocerotines the difference between the anterior and the posterior height in much less pronounced (Fig 23D–E). In most cases in the literature, only the maximal height is provided, without stating where it was measured, which complicates direct comparisons (Fig 23). In *Aceratherium incisivum* from Höwenegg (Germany) this ratio (70–73.4%, n = 4) is close to that of ‘*C.*’ *wimani*, which shows the least degree of shortening of the limbs among any chilothere (Fig 23). In the European teleoceratine *Brachypotherium brachypus* the ratio (58.7–60.5%, n = 4, based on [85] and own data) is close to that of the chilotheres. This is also expressed in its morphology, which is much closer to that of *C. schlosseri* (compare Fig 23B, C). Though, the posterior tuberosity is not as strongly developed in *Brachypotherium brachypus* from Steinheim (Fig 23C). In the American short-limbed hornless rhinocerotid *Teleoceras*, the scaphoid seems to be similarly compressed (50–55%, n = 3) as in *Chilotherium orlovi* [86], which exhibits the lowest value of all chilotheres (Fig 23).

**Fig 24:**
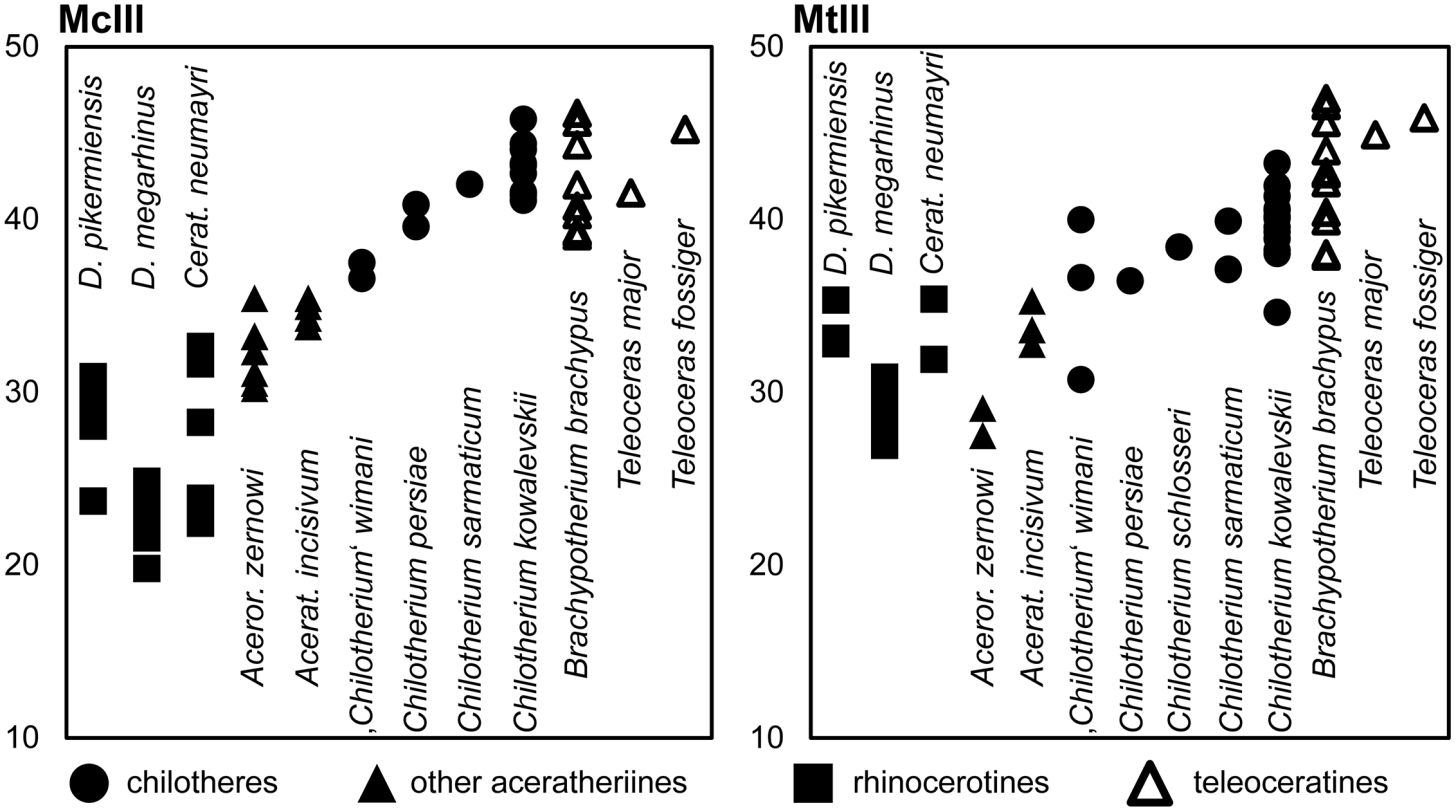
Metrical comparison of chilothere metapodials. Univariate diagrams of the proximal transversal diameter (DΤprox max) to length (L) ratio of the McIII and MtIII of chilotheres compared to other rhinocerotids. Measurements can be found in S1 Tables 10 and 22.

The most characteristic portion of the shortened limbs of chilotheres are the metapodials [7]. These are rather stout, especially when compared to other rhinocerotids (Fig 24) like the contemporaneous tandem-horned rhinocerotines *Dihoplus pikermiensis* and ‘*Ceratotherium’ neumayri* or the Middle Miocene aceratheriine *Acerorhinus zernowi* from Tung Gur (China) [75]. *Aceratherium incisivum* from Höwenegg [74] seems to be somewhat closer to *Chilotherium* than to *Acerorhinus zernowi*, especially concerning the dimensions of the MtIII (Fig 24). In the MtIII diagram specifically, it is visible that there is a broad range in the values of ‘*C.*’ *wimani*, which is however lower than that of *C. kowalevskii* for instance (Fig 24). The Middle Miocene European teleoceratine *Brachypotherium* exhibits similar ratios to the chilotheres (McIII: 39–46.1%, n=9, and MtIII: 38–47%, n=12 [85]), which overlap to an important degree with the ratios of *C. kowalevskii* (Fig 24). The American teleoceratine *Teleoceras* also exhibits similarly short or even shorter metapodials than *Chilotherium* [87], as indicated by the single data points for each species (Fig 24). A McIV (MNHN.F.TRQ329) from Küçükçekmece (Turkey) that was attributed to *C. schlosseri* [46], exhibits proportions that do not fit any chilothere. This McIV from Küçükçekmece is more slender (TDdia/L=22.3%) than the McIV of the chilothere compared herein (27.5–34.8%, n=14). Instead, its’ proportions fit much better those of *Acerorhinus zernowi* from Tung Gur (19.8–23.9%, n = 3) [75]. In the same locality, the presence of another hornless rhinocerotid, *Persiatherium* sp., has been reported, which is not a chilothere and most likely had a more elongated appendicular skeleton. Therefore, this McIV (MNHN.F.TRQ329) could possibly belong to *Persiatherium* instead of *Chilotherium*. The MtIII from the same locality (MNHN.F.TRQ335) that was also assigned to *C. schlosseri* was described as not bearing a cuboid facet. However, in all *C. schlosseri* specimens from Samos that preserve the relevant portion a cuboid facet is indeed present. Therefore, it is most plausible that the MtIII MNHN.F.TRQ335, does not belong to this species. On the other hand a McIV from Loc. 26 in the Sinap region referred to as ‘*Acerorhinus* sp. nov.’, has an index of 30.3% that falls well into the value range of the chilotheres (27.5–34.8%, n = 14) and the overall morphology of this bone also matches the morphology seen in *Chilotherium*. Therefore, it is most likely that this bone actually does belong to *Chilotherium* or another chilothere and not *Acerorhinus*.

**Fig 25:**
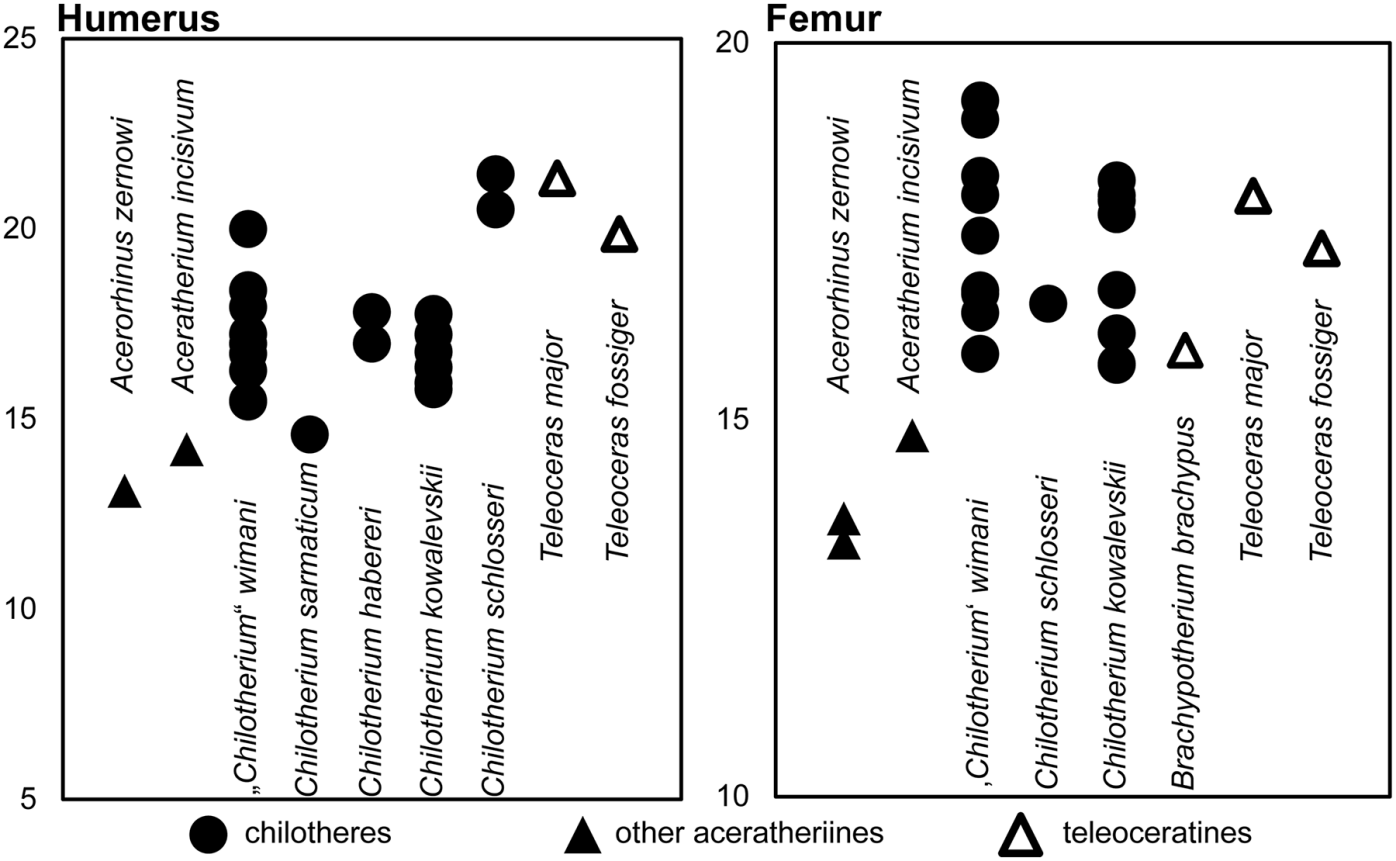
Metrical comparison of chilothere humeri and femora. Univariate diagrams of the transversal diameter of the diaphysis (TDdia) to length (L) ratio of the humerus and femur of chilotheres compared to other aceratheriines. Measurements can be found in S1 Tables 1 and 12.

The trend of shortening the limbs is also demonstrated in other bones of the appendicular skeleton. For instance, the humeri and femora in chilotheres are also relatively stouter than in other aceratheriines, like *Aceratherium incisivum* and *Acerorhinus zernowi* (Fig 25). Within rhinocerotids only teleoceratines have reached a similar degree of limb shortening, as exemplified by *Teleoceras major* and *Teleoceras fossiger* in Fig 25. Although, in the Middle Miocene European teleoceratine *Brachypotherium brachypus* the femur is not quite as shortened (15.8%, n = 1 [85]), still being comparable to the lowest values of *Chilotherium* (Fig 25).

Within chilotheres there seems to be a gradual increase in size and further shortening of the limbs. More specifically, *E. samium* and ‘*C.*’ *wimani*, which were considered as more “primitive” chilotheres, are somewhat smaller based on the dental and cranial material [e.g., 10]. This is also reflected in the appendicular skeleton, as confirmed by the comparison of the published postcranial data of ‘*C.*’ *wimani* from the Linxia Basin (China) to that of other chilotheres (Figs 23–25, S1 Table). The only other well-sampled chilothere that seems to be comparable in size is the chronologically oldest European representative, *C. sarmaticum* from the Vallesian of Berislav (Ukraine) [11,45]. Whereas the younger, middle to late Turolian chilotheres *C. schlosseri* from Samos (Greece) and *C. orlovi* from Pavlodar (Kazakhstan) seem to be the largest chilotheres [84], while at the same time *C. orlovi* also seems to be among the species with the most pronounced shortening of appendicular skeleton. The only chilothere of younger geological age is *Shansirhinus*, which is, however, exclusively known from craniodental material. For the longest time, only teeth were known from this species [24,73,88] and only recently complete skulls were described from Upper Miocene and Lower Pliocene deposits from China [76,89,90], making it the youngest reported representative of the chilotheres until now and the only chilothere surviving into the Pliocene. Nonetheless, no postcranial material has been described for any *Shansirhinus* species to be used in our current comparisons.

The possible attribution of the aceratheriine material (a partial forelimb) from Loc. 12 of the Upper Miocene Sinap Formation in Turkey [81: fig. 12.15] to the genus *Chilotherium* is herein rejected, due to the existence of a well-developed, functional, and not at all reduced McV. It is known that in *Chilotherium* the McV is represented by a small and sesamoid-like rudimentary bone similar to what is observed in extant rhinoceroses and also seen in the herein studied *C. habereri* material from Kutschwan. That is why *Chilotherium* has been widely considered as tridactyl [3,7,16]. The condition of the Sinap specimen is more similar to *Aceratherium incisivum* from Höwenegg, which also includes a well-developed McV [74]. The shape and size of the McV from Sinap and those from Höwenegg are extremely similar. Also, the other associated metacarpals from Loc. 12 of Sinap, are overall similar in proportions and form to the metacarpals of *Aceratherium incisivum* from Höwenegg. The gracility index of these bones (McII: 28.5%, McIII: 27.9%, and McIV: 25.8%) is very close to the values in *Aceratherium incisivum* from Höwenegg (McII: 27.2–27.6%, McIII: 29.2–29.6%, and McIV: 24.3–27.4%, n = 4). Nonetheless, the Sinap material, with a length of 122 mm in the McIII from Loc. 12 [81], is smaller than *Aceratherium incisivum* from Höwenegg (140–144 mm, n = 4) [74]. It is more likely that the associated forelimb from Loc. 12 represents a non-chilothere aceratheriine, possibly closely related to *Aceratherium incisivum*; however, solely based on such material it is currently impossible to attempt any taxonomic attribution.

Bringing together the information from the literature based on the craniodental material that can be used for the identification of the chilotheres species and combining it with the results of the current study of the postcranial material refines their differentiations. The current study showed that one of the most promising elements for taxonomic distinction may be the astragalus. More specifically, the arrangement of the articular facet for the calcaneum seem to differ in some species. In *C. anderssoni*, *C. habereri*, *C. persiae*, *C. kowalevskii*, and *C. orlovi* all articular facets are well separated from each other [7,11,12], similar to *Aceratherium incisivum* from Höwenegg (Fig 17). The species ‘*C.*’ *wimani* and *C. sarmaticum*, exhibit a connection between the sustentacular facet, the cuboid facet and the distal calcaneal facet (Fig 17) [11,16]. In *C. schlosseri*, the condition of these articular facets varies intraspecifically, with two astragali (GMM 571 and NHMW-GEO-1911/0005/0424) exhibiting a wide connection between the sustentacular facet and the ectal calcaneal facet, which is not observed in any other chilothere species (Fig 17B). The astragalus morphology of *C. schlosseri* further differs from the other chilotheres by having a connection between the medial lip for the articulation of the tibia and the articular facet for the navicular (Fig 16K). This character is observed in all available astragali of *C. schlosseri*; in other chilotheres there is a well-developed groove separating the medial lip and the navicular facet. Thereby it can be assumed that these features are apomorphic for *C. schlosseri* and can be added to the diagnostic characters for the species.

The studied axes of *C. persiae* and *C. schlosseri* also differ slightly from each other (Fig 2). The ventral crest of the axis is wider and looks more massive in *C. schlosseri*, whereas in *C. persiae* it seems compressed in its’ middle. The posterior articular facet for third cervical vertebra has a high-oval shape with a slight, dorsal indentation in *C. schlosseri*, whereas in *C. persiae* it is more rounded, and the dorsal surface is flat. Additionally, in ventral view there is an indentation on the posterior side in *C. persiae*, whereas *C. schlosseri* does not feature such an indentation. Lastly, the area where the lateral walls of the neural canal of the axis connect to the vertebral body seems to extend more posteriorly in *C. schlosseri* than in *C. persiae*. Due to the small number of specimens, it is not possible to assess the intraspecific variability of these characters or whether they are also present in other species. In the scapula, the shape of the medial tuberosity, is more rounded in *C. persiae* and *C. schlosseri* and rather flattened in *C. habereri* but seems to be more protruding in *C. schlosseri* (Fig 3). In the humerus, the dimensions of the species seem to be most informative, since ‘*C.*’ *wimani* and *E. samium* have the smallest humeri [14,16], *C. orlovi* has the largest humeri [12] and most other species, *C. persiae*, *C. habereri*, *C. schlosseri*, and *C. kowalevskii*, have humeri with intermediate dimensions. Additionally, *C. schlosseri* exhibits the most proximodistally compressed humerus among the compared species, being comparable to *Teleoceras* (Fig 25).

The patella also could be helpful for the taxonomic separation of chilotheres (Fig 13). The current comparison showed that there are two different morphotypes in the patella of chilotheres. The probably plesiomorphic ‘*C.*’ *wimani* has a more proximodistally elongated patella than *C. habereri* and *C. persiae*. In the latter two species, the patella is much wider. In the case of *C. persiae*, the wider form of the patella corresponds to the mediolaterally oriented tuberosity in the proximal epiphysis of the tibia. If this is true, it would mean that *C. schlosseri*, that has a more anteroposteriorly oriented tuberosity in the tibia, would have a patella that is more similar to that of ‘*C.*’ *wimani*. Besides the significance for taxonomy and phylogeny, patellar morphology is known to be associated with ecological adaptations [91]. In primates for instance, it has been shown that a proximodistally elongated patella is associated with running, climbing, and leaping behaviour, whereas the wider patella found in great apes probably reflects a more mobile knee joint [e.g., 92,93]. Recently, it was suggested that in extant Perissodactyla patellar morphology is rather conservative, being correlated to their phylogenetic affinities [83]. Chilotheres seem to be an exception to this rule, and the patellar morphology may well be informative also for their ecology, with species like ‘*C.*’ *wimani* that have a more proximodistally elongated patella being potentially somewhat more cursorial. However, additional material and analyses are needed to confirm such correlations.

Another postcranial element that seems to offer some taxonomic insight is the tibia. The dimensions of the bone already seem to help in the identification of the taxon, as some species like ‘*C.*’ *wimani* are fairly small. More interestingly, the morphology of the lateral tuberosity of the proximal articulation, for the attachment of the patellar ligaments, is differently shaped in *C. schlosseri* and *C. persiae* (Fig 14C, G). In proximal view, in the former one it is anteroposteriorly oriented, being higher than wide, and in the latter one it is more mediolaterally oriented being wider than high. Unfortunately, the proximal view of the tibia is not provided for any other chilothere, therefore it is not possible to assess the state of this character in any other species.

Lastly, metapodial bones can be useful for the separation of some species. Their dimensions overall, and most prominently the length, are diagnostic. Korotkevitch (1970) [11], showed that *C. sarmaticum* from Berislav has shorter metapodials than *C. kowalevskii* from Grebeniki, based on the comparison of his own measurements to those provided by Krokos (1917) [11,17]. However, this study did not take into account data available from other localities such as Samos [14], Odessa [36], and the Red Clay localities from China [7]. Additionally, since then, there have been some new studies that have described postcranial material of chilotheres [12,16,46,81], which need to be considered. The combined comparison of the data from the literature and the newly studied material of *C. persiae* from Maragheh, *C. habereri* from Kutschwan, and *C. schlosseri* from Samos does not directly confirm the assumption of Korotkevitch (1970) [11], but shows that the chilothere species exhibit varying degrees of shortening of the limbs. Instead of *C. sarmaticum* having significantly shorter metapodials, it seems that the degree of shortening is similar, possibly even lower than that seen in *C. kowalevskii* (Fig 24). However, it seems that there is a general separation of chilotheres into two size classes. The large size group is represented by *C. kowalevskii*, *C. schlosseri*, *C. anderssoni*, *C. persiae*, and *C. orlovi*, whereas *C. sarmaticum*, ‘*C.*’ *wimani*, *Chilotherium* indet. from Kavakdere, and possibly *E. samium* have smaller dimensions. A similar observation was also noted by Bayshashov et al. (2024) [84], who mentioned three size groups, with *C. sarmaticum* representing the small one, *C. schlosseri* (considered as the senior synonym of *C. kowalevskii* by these authors) and *C. anderssoni* representing the intermediate one, and only *C. orlovi* representing a distinctly larger size group. While *C. orlovi* seems to be the largest chilothere known to date, in our current study we divide the chilotheres in only two size groups, with *C. orlovi* being part of the large size group, for the purpose of this comparison. Additionally, there seems to be a temporal trend towards further shortening of the limb and becoming larger through time within the chilotheres. This is exemplified by the fact that the stratigraphically oldest chilotheres, like *C. sarmaticum* and ‘*C.*’ *wimani* from the Vallesian, are in fact the smallest and have relatively longer limbs compared to younger chilotheres like *C. schlosseri* and *C. orlovi* from the middle to late Turolian.

Overall, these new findings that are based on the postcranium confirm previous assumptions about chilotheres being characterised by shortened limbs compared to other rhinocerotids [e.g., 3,7,16]. The only comparable group that independently acquired a similar bodyplan is the Teleoceratini [e.g., 86,87]. When comparing chilotheres with other Eurasian rhinoceroses that lived during the Late Miocene, like ‘*Ceratotherium*’ *neumayri*, *Dihoplus pikermiensis*, and *Aceratherium incisivum* [27,74,94–96], it becomes clear that chilotheres differ significantly from the other members of the Rhinocerotidae and that despite often living in the same environment they were adapted to a different lifestyle. The results of our current study seem to also generally agree with what has been inferred from skull comparisons, concerning the relationship between different chilotheres. More specifically, it was already suggested that ‘*C.*’ *wimani* represents a more plesiomorphic chilothere, based on the very little separated parietal crests, the convex frontal region, the concave dorsal profile of the skull, the highly raised caudal portion of the skull, and the relatively simple tooth morphology [9,10,37]. This idea is corroborated by the comparatively small postcranial elements that are not quite as shortened as in other chilotheres (Fig 24). On the other hand, *C. schlosseri* was suggested to be a highly derived chilothere based on the strongly depressed frontal and nasal region of the skull, the generally flat and wide skull shape, and the complicated secondary enamel folds in the upper dentition [8,10]. This is further supported by the relatively large size and short appendicular elements, along with the derived morphology of the astragalus that differs from all other chilotheres.

Unfortunately, for many non-*Chilotherium* chilotheres, such as *E. samium*, *Shansirhinus* spp., and ‘*C.*’ *primigenium*, no postcranial material is available [10,14,37,76,89,90]. This makes it impossible to assess how widespread the shortening of the limbs was within the chilotheres. Nonetheless, the fact that ‘*C.*’ *wimani* – a species that is generally considered to be a “primitive” chilothere – exhibits shortened limbs in comparison to non-chilothere rhinocerotids, suggests that it is a plesiomorphic character within the chilothere group. It was recently suggested that ‘*C.*’ *wimani* should actually represent a different genus within the chilotheres, because it lacks one of the key autapomorphies of the genus, which is the depression in the frontal region of the skull, similar to *E. samium* and ‘*C.*’ *primigenium*, and unlike *C. anderssoni* and *C. schlosseri* [10]. The results of our study also support the notion that ‘*C.*’ *wimani* differs from the typical species of *Chilotherium*. In the case where ‘*C.*’ *wimani* is removed from the genus *Chilotherium* sensu stricto, the shortened limbs may actually prove to be a shared feature within chilotheres in general.

## Conclusion

The postcranial material of the hornless rhinocerotid group Chilotheriina has received little attention throughout their research history. Most studies have focussed on the skull and more specifically on the dental morphology. Herein, we describe the postcranium of three chilothere species: *Chilotherium persiae* from the Upper Miocene of Maragheh (Iran), *Chilotherium habereri* from the Upper Miocene of Kutschwan (China), and *Chilotherium schlosseri* from the Upper Miocene of Samos Island (Greece). We compared the newly described material with respective material of other chilotheres that is available in the literature and found some characters that seem to be taxonomically informative, including features in the scaphoid, tibia, patella, astragalus, and metatarsals. Additionally, we pointed out features that vary between the species, but due to the limited material their significance can presently not be evaluated. The new findings confirm that the shortening of the limbs is a key feature shared within chilotheres and support assumptions about the relationships between some species that were based only on cranial material until now.

## Supporting information

S1 Table Measurements of the studied material and species compared from the literature. (XLSX)
